# The Alcohol Dehydrogenase Gene Family in Melon (*Cucumis melo* L.): Bioinformatic Analysis and Expression Patterns

**DOI:** 10.3389/fpls.2016.00670

**Published:** 2016-05-18

**Authors:** Yazhong Jin, Chong Zhang, Wei Liu, Yufan Tang, Hongyan Qi, Hao Chen, Songxiao Cao

**Affiliations:** ^1^Key Laboratory of Protected Horticulture of Education Ministry and Liaoning Province, Department of Horticulture, Shenyang Agricultural UniversityShenyang, China; ^2^College of Agriculture, Heilongjiang Bayi Agricultural UniversityDaqing, China

**Keywords:** melon, alcohol dehydrogenases, bioinformatic analysis, gene identification, expression pattern of gene

## Abstract

Alcohol dehydrogenases (ADH), encoded by multigene family in plants, play a critical role in plant growth, development, adaptation, fruit ripening and aroma production. Thirteen ADH genes were identified in melon genome, including 12 ADHs and one formaldehyde dehydrogenease (FDH), designated *CmADH1-12* and *CmFDH1*, in which *CmADH1* and *CmADH2* have been isolated in Cantaloupe. ADH genes shared a lower identity with each other at the protein level and had different intron-exon structure at nucleotide level. No typical signal peptides were found in all *CmADHs*, and *CmADH* proteins might locate in the cytoplasm. The phylogenetic tree revealed that 13 ADH genes were divided into three groups respectively, namely long-, medium-, and short-chain ADH subfamily, and *CmADH1,3-11*, which belongs to the medium-chain ADH subfamily, fell into six medium-chain ADH subgroups. *CmADH12* may belong to the long-chain ADH subfamily, while *CmFDH1* may be a Class III ADH and serve as an ancestral ADH in melon. Expression profiling revealed that *CmADH1, CmADH2, CmADH10* and *CmFDH1* were moderately or strongly expressed in different vegetative tissues and fruit at medium and late developmental stages, while *CmADH8* and *CmADH12* were highly expressed in fruit after 20 days. *CmADH3* showed preferential expression in young tissues. *CmADH4* only had slight expression in root. Promoter analysis revealed several motifs of *CmADH* genes involved in the gene expression modulated by various hormones, and the response pattern of *CmADH* genes to ABA, IAA and ethylene were different. These *CmADHs* were divided into ethylene-sensitive and –insensitive groups, and the functions of *CmADHs* were discussed.

## Introduction

Alcohol dehydrogenases (ADH, EC 1.1.1.1) belong to dehydrogenase enzymes superfamily, and are widely distributed in all types of organisms (Chase, 1999; Jönvall et al., 2010; Strommer, 2011; Alka et al., 2013). ADH enzyme has long been the subject of molecular studies and encoded by multigene family in eukaryotic and prokaryotic kingdoms, catalyzing the interconversion between alcohols and aldehydes (Höög et al., 2003; Thompson et al., 2007). In humans, animals, yeast and bacterias, ADHs have been widely investigated (Khan et al., 2010; Kumar et al., 2012; Alka et al., 2013; Çelik and Aktas, 2013; Jönvall et al., 2013; Plapp et al., 2013; Quaglia et al., 2013), and ADH genes were involved in an astonishingly wide range of metabolic processes (Höög et al., 2003; Thompson et al., 2007; Strommer, 2011**)**. Hence, these ADH genes were classed into several main superfamilies respectively, namly medium- (∼350 amino acid residues), short- (∼250 residues) and long-chain ADH or Iron-ADH genes superfamilies (600–750 residues or approximately 385 amino acid residues up to almost 900 residues) (Chase, 1999; Deng et al., 2002; Alka et al., 2013; Jönvall et al., 2013), and the medium-chain ADHs clustered into eight classes in vertebrates, based on sequence similarity, catalytic features and gene expression patterns (Thompson et al., 2007). But functional roles of these ADHs are not fully understood.

Sets of ADH genes or ADH-like genes have been identified in the genomes of poaceae, rosaceae, brassicaceae, fabaceae, and pinaceae plants (Thompson et al., 2010; Zheng et al., 2011). So far, most members of the ADHs in plants characterized at the gene level belonged to the medium-chain ADH protein superfamily (including ADH1, EC 1.1.1.1 and FDH, Class III ADH, EC 1.2.1.1; Chase, 1999), which usually contains zinc ligands in their active site (Tesnière and Verriès, 2000; Garabagi et al., 2005; ManrÍquez et al., 2006; Koutsompogeras et al., 2010; Singh et al., 2010; Komatsu et al., 2011; Pathuri et al., 2011; Bukh et al., 2012; Iaria et al., 2012; Min et al., 2012; Cheng F.F. et al., 2013), and the short-chain ADH protein superfamily, which lacks zinc-liganding cysteine residues in their coenzyme binding regions, and the molecular functions of only a few short-chain ADH genes were known (ManrÍquez et al., 2006; Gonzalez-Agüero et al., 2009; Kim et al., 2009; Strommer, 2011; Moummou et al., 2012). Other ADHs have been not noted in plants, such as long-chain ADHs or Iron-ADH genes. On the basis of phylogenetic analyses, the medium-chain ADH genes in plant were distributed in different subgroups (Perry and Furnier, 1996; Gaut et al., 1999; Small and Wendel, 2000; Komatsu et al., 2011; Zheng et al., 2011). Previous studies highlighted that the medium-chain ADHs in plant were involved in response to abiotic and biotic stress which induced the specific expression of these ADHs in different tissues of soybean, wheat and barley, implying that they may participate in different tissues development in stresses (Komatsu et al., 2011; Pathuri et al., 2011; Yamauchi et al., 2014). Moreover, regardless of medium- chain ADHs or short-chain ADHs, ADH genes have been shown to play a major role in fruit ripening and aroma synthesis (Tesnière and Verriès, 2000; ManrÍquez et al., 2006; Singh et al., 2010; Zhang et al., 2011; Zheng et al., 2011; Bukh et al., 2012; Iaria et al., 2012; Moummou et al., 2012). However, the actual participation of medium-chain ADHs in aroma volatile production *in vivo* has been only clearly demonstrated in tomato fruit by over-expressing or down-regulating the *LeADH2* gene (Speirs et al., 1998). Other detailed studies related to role of ADH genes (including medium- and short-chain ADHs) in relation to aroma synthesis have been proved in tomato, melon and mango by recombinant protein (ManrÍquez et al., 2006; Singh et al., 2010; Moummou et al., 2012). In addition, persimmon *DkADH*, Artemisia annua *AaADH2*, Sedum sarmentosum *SsADH* (belonging to medium-chain ADH family) and other short-chain ADHs have been implicated in other primary and secondary metabolisms (Polichuk et al., 2010; Tonfack et al., 2011; Sung et al., 2012). In panax ginseng, the expression pattern of *PgADH* under abiotic stimuli suggested that a short-chain ADH, *PgADH*, was involved in responses to hormone-related environmental stresses (Kim et al., 2009). Meanwhile, the tissue-specific patterns of these ADH gene expressions among different tissue under various environmental stresses and during fruit ripening and aroma synthesis also provided a few evidences of functional specialization. We conclude further investigation is required to make functional annotation for the majority of predicted ADH in plant genomes.

In melon, two ADH genes, *CmADH1* and *CmADH2*, have been identified and characterized (ManrÍquez et al., 2006). The release of melon genome provides an easy way to identify new members of ADH gene family. This study is a continuation of CAD genes (belonging to dehydrogenase enzymes superfamily) research in melon (Jin et al., 2014). In this study, we identified 12 *CmADHs*, including *CmADH1* and *CmADH2*, and one FDH gene from the melon genome, and compared ADH sequences from a wide variety of plants, making full use of the available plant genome sequences (*Arabidopsis*, mango, tomato, grape et al.). Alignment and phylogenetic analysis of the ADH gene family with related ADH proteins from other species indicated that these ADH genes were classified into three groups and the medium-chain melon ADHs were clustered into six subgroups separately. We analyzed the structure and the promoter of melon ADH genes, and also investigated these ADH genes transcripts in response to various fruit development stages and various plant hormones, and monitored tissue-specific expression. We reported here the results of these analyses, suggesting that the *CmADHs* may be involved in melon fruit development induced by various hormones and during development and ripening. Information gained from this investigation will significantly advance the understanding of the function of predicted ADH genes in Melon.

## Materials and Methods

### Plant Materials

All the experiments were conducted on a commercial oriental melons (*Cucumis melo* var. *makuwa* Makino) cultivar, ‘CaiHong7.’ All melon plants were grown in a greenhouse under standard cultural practices at Shenyang Agricultural University, Shenyang, China, from March to June in 2012, and the melon fruit were harvested at different development stages (1, 5, 10, 15, 20, 25, 30, 33, 36, 39, 42, 45, and 48 days after anthesis) for expression analysis as previously described (Jin et al., 2014). Physiological maturity of this melon is about 36 days after anthesis. To allow analysis of tissue-specific gene expression, mature leaf, developing leaf, pistillate and staminate flower, young stems and root tissues were collected from melon plants grown in a greenhouse, then frozen with liquid nitrogen and kept in -80°C refrigerator for further use.

### Hormone Treatments

Mature unripe oriental melons were harvested at 30 days after anthesis (pre-climacteric stage) with the same node of the plant at a mature green stage, before the onset of ripening. No blemished or diseased Fruits were chosen for treatment. Exogenous ethylene (100 μL/L), 1-methylcyclopropene (1-MCP, an ethylene perception inhibitor) (100 μL/L) and control (water) treatments were performed according to our previous report (Jin et al., 2014). Then melon fruit of three treatments were allowed to ripen for 12 days at 23°C in air only. Three replications were carried out for treatments, and each replicate consisted of 15 fruits unless indicated otherwise. Fresh tissue was sampled every 48 h, frozen in liquid nitrogen and stored at -80°C until further use. Furthermore, for abscisic acid (ABA;100 μM) and auxin (IAA;100 μM) treatment, the fruit disks were dipped in a solution containing ABA or IAA in 0.2% teepol (detergent) and vacuum infiltrated for 2 h as described previously (Jin et al., 2014). Infiltrated melon fruits with 0.2% teepol were used as control. After treatment, fruit flesh were frozen in liquid nitrogen and stored at -80°C. Three replications were carried out for treatments or control groups.

### Identification of Melon ADH Genes

The keyword ‘alcohol dehydrogenase’ was used to search for the melon ADH sequences from the melon (*Cucumis melon* L.) genome^1^ (Garcia-Mas et al., 2012). In addition, to confirm the accuracy of these genes, the predicted ADH-like gene sequences were compared to ADH proteins in other species by a BLASTp retrieve. Only those sequences with a high score (>200) were selected.

### Sequence Analysis

The sequence analysis of *CmADHs*, including ADH protein prediction, functional domains and promoter analysis, signal peptides and disulfide bond prediction and *CmADHs* subcellular localization prediction, were performed according to the method described by Jin et al. (2014). Multiple alignments were performed with other known plant ADH proteins using Clustal Omega program and GENEDOC. Based on the neighbor-joining method (minimum evolution criterion, bootstrap values performed on 1000 replicates), phylogenetic analyses of putative melon ADH proteins were performed using MEGA5^2^ program.

### RNA Isolation and cDNA Synthesis

Total RNA from fruit samples, leaves, stem, root, and flower material, were extracted by the method described by Jin et al. (2014), quantified by the NanoDrop spectrophotometer ND-1000 and checked for integrity by electrophoresis (28S rRNA/18S rRNA ratios). RNA was treated by DNAse I (Promega, Madison, WI, USA) at 37°C for 50 min, re-precipitated and concentrated (40 μL) to remove any trace of genomic DNA. cDNA synthesis was initiated from DNase I-treated RNA using M-MLV RTase cDNA Synthesis Kit following the manufacturer’s instructions (Cat#D6130, TaKaRa, Tokyo, Japan).

### Semi-quantitative PCR and Real-Time PCR

For semi-quantitative PCR and real-time PCR, gene-specific oligonucleotide primers for each ADH gene were designed by Primer3^3^ and were listed in **Table 1**. The specificity of each pair of primers was determined by agarose gel electrophoresis and PCR products resequencing, then semi-quantitative PCR and Real time PCR were performed for gene expression studies according to the method described by Jin et al. (2014). *18S*rRNA DNA fragment (148 bp) of melon as an internal control.

**Table 1:** Semi-quantitative PCR and Real-time PCR primers.

Gene	Primer	Sequence (5-3)
*CmFDH1*	CmFDH1-F	GACATTTGAGTTAGGATGG
	CmFDH1-R	GGTGATAGTTACAATCTTGG
*CmADH1*	CmADH1-F	CTAATGAAGTCCGATTGAAG
	CmADH1-R	ATGATCTCCTGGTTGAAG
*CmADH2*	CmADH2-F	GCCATTGTTGATTCACTCA
	CmADH2-R	CATTCGCAGTCACTTGTAA
*CmADH3*	CmADH3-F	GCTGGTGTCCATCGCTGTT
	CmADH3-R	CCAACTCCGCTTGGTAATGG
*CmADH4*	CmADH4-F	ACCACCACAAGCCAATGAAG
	CmADH4-R	TGACGACATTCTCCACATTCC
*CmADH5*	CmADH5-F	GGACTCAACTTCCTCATT
	CmADH5-R	GTAACTCCGTAGAAACATAC
*CmADH6*	CmADH6-F	CAGAACTCTTGGCGATAA
	CmADH6-R	TGGTCTACTCCTAACACTA
*CmADH7*	CmADH7-F	CACACTATTCACTGGAGA
	CmADH7-R	CCACGGTATATTCACTGA
*CmADH8*	CmADH8-F	CCAATCATCCATCTGTATC
	CmADH8-R	GGTTAATGCGAGTAAGTG
*CmADH9*	CmADH9-F	TCCGATCTTCCAATGCTTAT
	CmADH9-R	ATGAGGCAACGAAGTGAT
*CmADH1*0	CmADH10-F	TCAGATATTCCCACTCTTCT
	CmADH10-R	ACAACACAACCGATGATG
*CmADH1*1	CmADH11-F	CACTGGTAATTGAGGAAG
	CmADH11-R	CTACACTCTCCACAACTA
*CmADH1*2	CmADH12-F	TATGGAGGATTAGACATCTG
	CmADH12-R	CCTTATCGCAAGTTGAGT
*18srRNA*	18srRNA-F	AAACGGCTACCACATCCA
	18srRNA-R	CACCAGACTTGCCCTCCA

### Statistical Analysis

Data are expressed as mean values ± standard deviation of three independent experiments (*n* = 3). The data were analyzed by the analysis of variance (ANOVA) using the SPSS 13.0 statistics program, and statistical significance of differences was were calculated by a one-way ANOVA following Duncan’s multiple range tests for each experiment at a *P* < 0.05 level. Origin 8.0 (OriginLab, Northampton, MA, USA) was used to draw the figures.

## Results

### Identification of Melon ADH Genes

All the predicted ADH genes in the melon genome were collected and compared with ADH genes in other species. Hence, the presence of functional domains was checked via NCBI’s Conserved Domain Database (CDD)^4^, and 13 genes (including 12ADHs and 1 FDH), encoding full-or nearly full-length functional proteins, were identified in the melon genome.

By ExPASy tools, we found that the longest ADHs protein consisted of 635 amino acid residues, and the shortest consisted of 266 amino acid residues. The ORF length ranged from 801 to 1908 nucleotides. The predicted molecular weight and isoelectric points of all ADHs proteins ranged from 29.0 kDa/5.19–68.5 kDa/9.06, respectively (**Table 2**). Of these ADH genes, two of them have been reported previously and were labeled with their published names (ManrÍquez et al., 2006) (**Table 2**). For the other 11 unreported genes, we gave them names by adding a number to their family name in the order which they were searched, in which one ADH was named as *CmFDH1* on account of the presence of same functional domains with reported FDH genes in other species (Dolferus et al., 1997).

**Table 2:** The information of ADH genes in melon.

Gene	Gene accession No.	Published name	ORF length	amino acid (aa)	Melon predicted protein No.	Molecular weight (kD)	Location	Isoelectric Point
*CmFDH1*	MELO3C022399T1		1014	337	MELO3C022399P1	36.18	CM3.5_scaffold00052	6.74
*CmADH1*	ABC02081	*CmADH1*	1140	379	ABC02081.1	41.01	CM3.5_scaffold00061	6.83
*CmADH2*	ABC02082	*CmADH2*	801	266	ABC02082.1	29.09	CM3.5_scaffold00023	8.72
*CmADH3*	MELO3C026552T1		1239	412	MELO3C026552P1	45.02	CM3.5_scaffold00096	7.38
*CmADH4*	MELO3C027151T1		1146	381	MELO3C027151P1	41.44	CM3.5_scaffold00483	5.55
*CmADH5*	MELO3C005792 T1		1179	392	MELO3C005792 P1	42.24	CM3.5_scaffold00005	6.17
*CmADH6*	MELO3C026553 T1		1164	387	MELO3C026553 P1	42.02	CM3.5_scaffold00096	5.88
*CmADH7*	MELO3C002189 T1		1143	380	MELO3C002189 P1	40.67	CM3.5_scaffold00001	6.55
*CmADH8*	MELO3C003251 T1		1272	423	MELO3C003251 P1	45.81	CM3.5_scaffold00002	8.62
*CmADH9*	MELO3C011043 T1		1104	367	MELO3C011043 P1	40.29	CM3.5_scaffold00014	5.19
*CmADH10*	MELO3C026554 T1		864	287	MELO3C026554 P1	42.04	CM3.5_scaffold00096	6.61
*CmADH11*	MELO3C023687 T1		984	327	MELO3C023687 P1	36.59	CM3.5_scaffold00061	6.76
*CmADH12*	MELO3C019503 T1		1908	635	MELO3C019503 P1	68.51	CM3.5_scaffold00038	9.06

### Intron-Exon Structure of Melon ADH Genes

**FIGURE 1:**
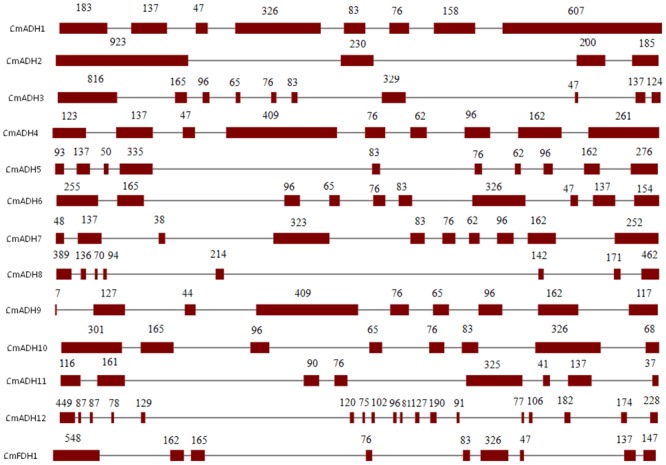
**Intron-exon structures of ADH genes from melon.** Exons and introns were indicated by open boxes and lines respectively. Numbers above boxes indicated the exon sizes. The intron sizes were not to scale. The names of ADH genes and intron-exon structure were indicated at the left and right sides respectively.

Structural analysis of the identified melon ADH genes revealed different intron-exon patterns both in relation to position and number of introns which ranged from 2 to 17 per gene (**Figure 1**). Furthermore, the substantial differences in the size between the exons were observed. The standard number of introns in plant ADH genes in the common ancestor is 9, located at equivalent positions in ADH genes throughout the plant kingdom (Strommer, 2011). However, plant ethanol-active ADH genes have evolved from class III ADH genes by gene duplication and acquisition of new substrate specificities (Dolferus et al., 1997). Based on **Figure 1**, *CmFDH1* (Class III ADH;GSH-FDH) contained nine exons and eight introns, while the intron-exon structures of other 12 *CmADHs* were relatively complex. *CmADH1, CmADH8, CmADH10* and *CmADH11* had seven introns, and *CmADH3, CmADH5, CmADH6* and *CmADH7* had nine introns, and *CmADH4* and *CmADH9* contain eight introns; *CmADH2* had two introns, moreover *CmADH12* contained 17 introns (**Figure 1**). For ADH-plant genes, introns patterns contributed more evidences for gene acquisition through duplications, insertion or loss of introns (Charlesworth et al., 1998; Small and Wendel, 2000; Strommer, 2011). Additionally, both 76-bp and 83-bp sequence were found in six sequences, and a 76-bp sequence was found in these sequences, except *CmADH2,8,12* (**Figure 1**). Nevertheless, it was unclear whether these sequence encoded the putative functional region of ADH genes.

### Sequence Characterization of CmADH Genes

**FIGURE 2:**
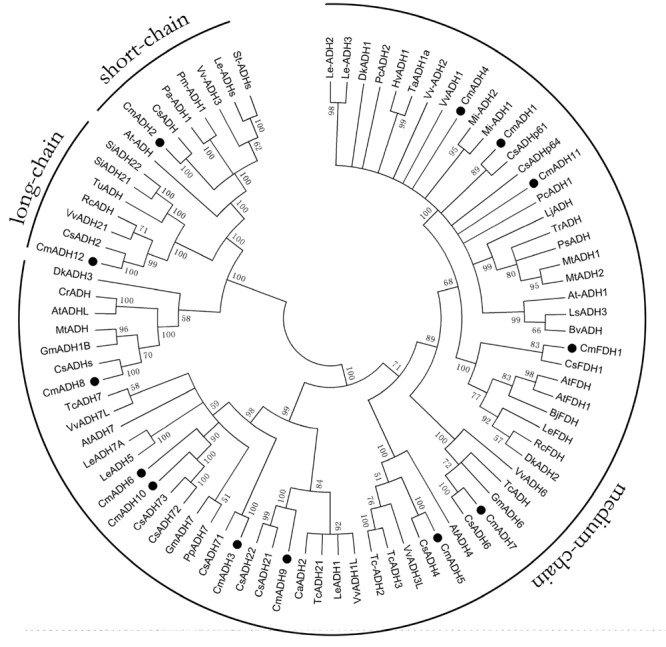
**Phylogenetic relationship among *CmADHs* and ADHs from other plant species.** The amino acid sequences were aligned by the Clustal Omega program, and the neighborjoining tree was drawn with MEGA5. The corresponding GenBank and the melon genome (https://melonomics.net/) were noted in the phylogenetic tree, and the accession number in the melon genome were *CmADH1* (MELO3C023685P4), *CmADH2* (MELO3C014897P1), *CmADH3* (MELO3C026552P1), *CmADH4* (MELO3 C027151P1), *CmADH5* (MELO3C005792P1), *CmADH6* (MELO3C026553P1), *CmADH7* (MELO3C002189P1), *CmADH8* (MELO3C003251P1), *CmADH9* (MELO3C0110 43P1), *CmADH10* (MELO3C026554P2), *CmADH11* (MELO3C023687P1), *CmADH12* (MELO3C019503P1), and *CmFDH1* (MELO3C022399P1). The number for each interior branch was the percentage of bootstraps value (1000 replicates). Black circle denoted 13 *CmADHs*. ADHs from other plants in our paper were in supporting information (Supplementary Table S3).

To understand the possible functional divergence of the individual members of melon ADH genes superfamily, the sequences of a set of ADH family members from other plant species, including cucumber, *Arabidopsis*, grape, potato, tomato, mango, apricot, peach, soybean, cocoa, were analyzed using a Neighbor**-**joining (NJ) phylogenetic tree by MAGA5. As shown in **Figure 2**, the phylogenetic tree divided these ADHs into three clades, namely medium-, short-, and long-chain ADHs, and the medium-chain ADHs in melon were clustered into six medium-chain ADHs subgroups, called as midum-chain groups I–VI, based on their amino acids sequence (**Supplementary Figure S1**). In Cantaloupe, *CmADH1* and *CmADH2*, two highly divergent genes, have been isolated, and it has been characterized that *CmADH1* belonged to the medium-chain zinc-binding type of ADHs and *CmADH2* was positioned in the short-chain ADHs (Singh et al., 2010). Moreover, C*mADH3-11* belonged to medium-chain zinc-binding type of ADHs and have not been characterized, in which *CmADH1, Pa-ADH1* (apricot) and *Le-ADHs* (tomato), having been characterized, were involved in fruit-ripening and aroma volatiles biosynthesis (ManrÍquez et al., 2006; Gonzalez-Agüero et al., 2009; Moummou et al., 2012). *CmADH12* belonged to long-chain ADHs which have not been characterized in plant; *CmFDH1* clustered into medium-chain zinc-binding type of FDHs, known as Class III ADH, and *AtFDH1* (*Arabidopsi*) and *OsFDH1* (rice) have been cloned (Dolferus et al., 1997); In addition, it is now recognized that FDHs are involved in plant development and stress response (Feechan et al., 2005; Rustérucci et al., 2007; Lee et al., 2008; Espunya et al., 2012).

**FIGURE 3:**
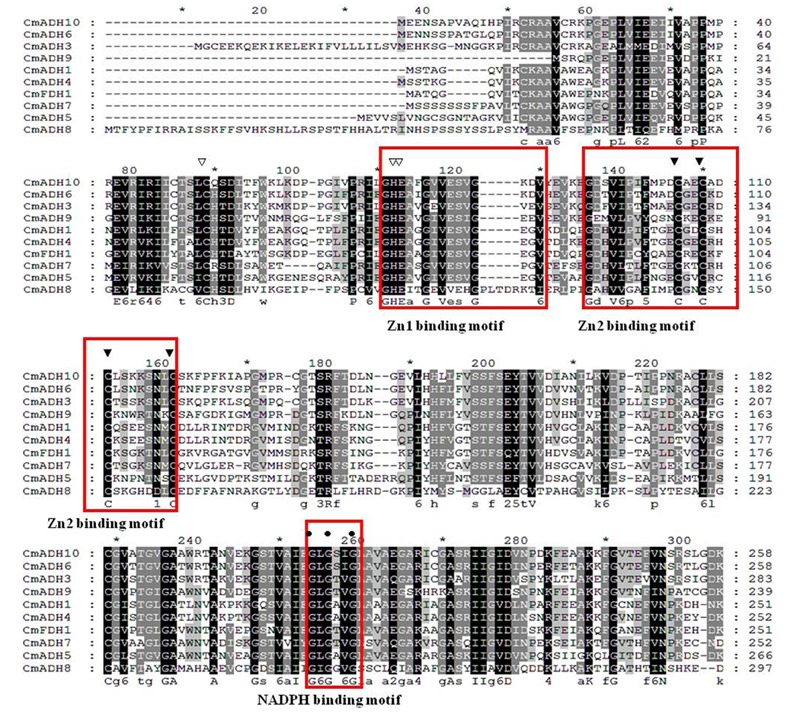
**Alignment of amino acid sequences of *CmADHs*.** Conserved important regions identified previously were marked as follows: white arrows denoted catalytic zinc ion coordinating residue, black arrows denoted structural Zn ion coordinating residue, the black circle denoted key residues for substrate specificity. The black square denoted key Phe299/Gly(300) residues for substrate specificity. The white square denoted key Trp199 and Asp123 residues for substrate binding. Locations of the Zn1, Zn2, and NADPH binding domains were shown in boxes. The alignment was performed with the Clustal Omega program.

Multiple alignments revealed that the *CmADH* genes showed lower identity in protein level with each other, as noted previously in Cantaloupe (ManrÍquez et al., 2006). Among the medium-chain zinc-binding type of *CmADHs*, there was lower identity between *CmADH1* and other medium-chain zinc-binding *CmADHs* in the range of 30–88.51% (**Figure 3**), whereas *CmADH1* showed the highest sequence identities to medium-chain ADH proteins from cucumber, mango, grape, tomato and *Arabidopsis* in range of 80–96.08% (**Supplementary Figure S2**), and other medium-chain *CmADHs* also showed higher sequence identities with other plants (**Supplementary Figures S3–S9**). All of the medium-chain zinc-binding *CmADHs* had the highly conserved Zn1 -binding signature [GHE(X)2G(X)5G(X)2V], the Zn2 structural motif [GD(X)9,10C(X)2C(X)2C(X)7C], and the NADPH-binding domain [GXG(X)2G] motif (so-called Rossmann fold) (**Figure 3**; **Supplementary Figures S2–S9**), which was the same with that of *CmADH1* proved (Singh et al., 2010), suggesting that these proteins appear to be zinc-dependent ADHs and members of the plant ADH protein family and the medium-chain dehydrogenase/reductase (MDR) superfamily (Persson et al., 1991; McKie et al., 1993). Nevertheless, *CmADH11*, a medium-chain melon ADH, lacked the key determinant residues of NADPH-binding domain at protein level (**Supplementary Figure S9**).

Furthermore, *CmADH2*, a short-chain ADH protein, and *CmADH12*, a long-chain ADH protein, had highly homology with corresponding ADHs from other plants (**Supplementary Figures S10** and **S11**). The sequence alignment showed that the *CmADH12* protein sequence was found most similar to corresponding ADH-like proteins from other plants, with identities close to 95.43% (**Supplementary Figure S11**), and contained an N-terminal cofactor binding pattern of typically TGXXXGXG and an active-site pattern of YXXXK which are two conserved regions around amino acids of short-chain dehydrogenases in plants (Joërnvall et al., 1999). Additionally, we used the protein database in NCBI as a source of amino acids sequence bioinformatics for *CmADH12*, and found that *CmADH12* possessed a coenzyme (NADPH) binding domain AAAGGTG, suggesting that *CmADH12* may belong to long-chain ADH subfamily, but may have the same catalytic function as shot-chain dehydrogenases. Interestingly, the analysis of the amino acids sequence of *CmADH12* showed that it did not contain structurally and functionally important iron-binding motif (G-X-X-H-X-X-A-H-X-X-GX-X-X-X-X-P-H-G), and NAD(H)-or NADP(H)-binding fingerprint patterns G-X-G-X-X-G or G-X-G-X-X-A, GGGSXXD and A/GXXATGG, and Trp docking motif (A-x-[DN]-x-x-T-G-[DEK]-x-x-W), possessed in long-chain or iron-dependent ADH (group III ADH) protein of prokaryotes, human and other mammals (Inoue et al., 1989; Deng et al., 2002; Lyon et al., 2009; Elleuche et al., 2013, 2014). Additionally, Filling et al. (2002) described several conserved residues of short-chain dehydrogenase, involved in coenzyme binding and catalysis, such as Thr12, Asp60, Asn86, Asn87, Ala88, Gly89, Gly131, Gly183, Asn111, Ser151 and Tyz157. In melon, *CmADH2* only contains five residues of these conserved residues, namely Thr12, Asp60, Gly183, Ser151 and Tyz157 (**Supplementary Figure S10**). These findings suggested that the biological functions of plant short-chain ADH proteins might be related to the diversity in amino acid sequence.

No typical signal peptides were found in all *CmADHs* after analyzing their N-terminals using the SignalP software. *CmADHs* subcellular localization prediction showed that these ADH genes might exist in the cytoplasm (Supplementary Table S2). Moreover, the transmembrane topology predictions of 13 *CmADHs* using TMHMM 2.0 software and ABTMpro showed that there was no internal transmembrane segment in *CmADHs*. We also found that there were 4 and 3 disulfide bonds in 12 melon ADH proteins by Scratch Protein Predictor, respectively, but there were 6 disulfide bonds in *CmADH12* protein.

### Promoter Sequence Analysis

Analysis of promoter sequences for the melon ADH genes allowed us to identify several motifs that were known to be involved in the regulation of gene expression in various developmental and physiological processes. A promoter motif search showed that *CmADHs* promoter contained putative regulatory elements corresponding to known *cis*-elements of eukaryotic genes (Barakat et al., 2009; Kim et al., 2010; Cheng H. et al., 2013). In melon ADH genes promoter, there were mainly two kinds of motifs, namely, *cis*-acting element involved in defense and stress responsiveness (such as hypoxia stress, heat stress) and *cis*-acting regulatory element involved in the response to various hormones, such as auxin (IAA), ethylene (ETH), ABA, salicylic acid (SA), and methyl jasmonate (MeJA) (Supplementary Table S1) (Barakat et al., 2009). Nevertheless, the most abundant motifs detected were the cis-elements involved in the response to biotic and abiotic stresses, including TC-rich repeats, LTR, W-BOX, ARE, HSE and MBS (Jin et al., 2014). At the same time, we still found that there were putative *cis*-acting regulatory elements involved in the MeJA-responsiveness, namely CGTA-motif, TGACG-motif and CCAAT-motif (Matton et al., 1990; Prashant et al., 2012; Cheng H. et al., 2013). TCA-element clustered in the promoters of *CmFDH1, CmADH1, CmADH3, CmADH5, CmADH7-9* and *CmADH12*, and **S**ARE were also present in *CmADH3* promoter, both of which were *cis*-acting regulatory element involved in the SA-responsiveness (Prashant et al., 2012; Cheng H. et al., 2013). ERE, ethylene-responsive element, was found in the promoter of *CmFDH1, CmADH1, CmADH2, CmADH3, CmADH5, CmADH6, CmADH8* and *CmADH12.* TGA (Auxin responsiveness), ABRE (ABA responsiveness), p-box and GARE (gibberellin responsiveness) were also present in several *CmADHs* promoter. Additionally, promoter sequence analysis revealed several conserved motifs, such as MBS-I and MBS-II (MYB binding site involved in flavonoid biosynthesis and drought-inducibility), HSE (heat stress responsiveness) and LTR (Low temperature responsiveness) (Prashant et al., 2012). *CmFDH1, CmADH3* and *CmADH9* possessed elicitor responsive element and enhancer. These results indicated that transcriptional regulation of these *CmADH* genes might be involved in fruit development and ripening and senescence and in the response to various stresses.

### *CmADHs* Expression in Vegetative Tissues

**FIGURE 4:**
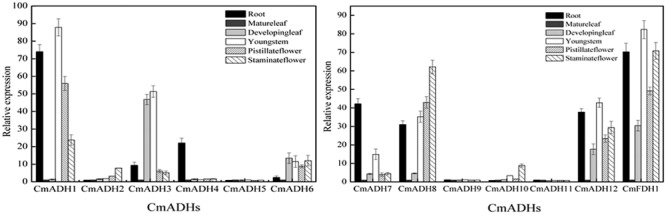
**Transcript levels of *CmADHs* in different melon organs.** Gene expressions of *CmADHs* in different organs in melon plants were determined by real-time PCR in root, developing leaves, mature leaves, young stems, pistillate flower petals and staminate flower petals in melon plants. *18s* was used as internal control. The expression level of the genes in mature leaves was set as “1.0.” Data represented the means ± SD (*n* = 3) of three biological samples. The experiments were carried out in triplicate.

In order to investigate the transcript levels of these 13 *CmADH* genes in different organs and tissues in melon plant, we collected samples of root, developing leaf, mature leaf, young stem, pistillate and staminate flower petal. Expression analysis using semi-quantitative PCR and real-time PCR showed that 12 *CmADH* and one *CmFDH* were constitutively expressed in these parts, but greatly varied in different tissues, and there were differences in same tissues between *CmADHs* (**Figure 4**; **Supplementary Figure S12**). Of the 12 ADH genes identified in melons, *CmADH2* and *CmADH10* were expressed most abundantly in root, developing leaf, mature leaf, young stem, pistillate and staminate flower. Because of the higher expression of *CmADH2* and *CmADH10* seleted as “1,” real-time PCR analysis of *CmADH2* and *CmADH10* showed a lower relative expression level (**Figure 4**). The expression of *CmFDH1* was higher in root, young stem and pistillate flower. *CmADH9* and *CmADH11* were either not expressed or expressed at very low levels in these tissues. Of all analyzed tissues, the *CmADH4* gene was highly expressed in the root, and only rarely expressed in other vegetative organs. *CmADH1* expression was the greatest in root and young stem, and *CmADH3* was expressed in vegetative organs, with the highest expression in the young leaf and stem. Furthermore, *CmADH5, CmADH6* and *CmADH7* showed similar expression patterns between vegetative organs, with lower transcript levels, and without significant differences except root (*P* < 0.05). *CmADH8* and *CmADH12* were also expressed in all tissues except mature leaf, showing lower expression in young leaf.

### *CmADHs* Expressions during Melon Fruit Development

**FIGURE 5:**
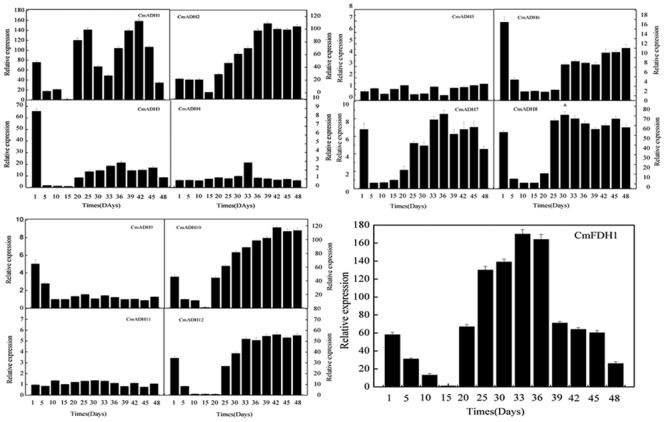
***CmADHs* relative expression in developing stages of melon fruit after anthesis were determined by real-time PCR.** *18s* was used as internal control. The expression level of *CmADHs* in melon fruit at 15 days after pollination was set as “1.0.” Data represented the means ± SD (*n* = 3) of three biological samples. The experiments were carried out in triplicate.

Transcript analysis indicated that these 12 *CmADH* genes and one *CmFDH* gene studied here were specifically expressed in fruit of different development stages, with remarkable differences in fruit (**Figure 5**; **Supplementary Figure S12**). The pattern of changes in transcript levels was similar for *CmADH1, CmADH3, CmADH8, CmADH12* and *CmFDH1* from 1 to 15 days after anthesis with gradual or sharp decrease at 15 days. While the expression of *CmADH3, CmADH8* and *CmADH12* showed an increase after 15 days, and the levels of these 3 ADH genes transcript were always maintained up to 48 days after anthesis. However, *CmADH1* had higher expression in fruit during development, except from 30 to 32 days and 48 days after anthesis. In contrast, *CmADH2* and *CmADH10* were consistently expressed with an increase in transcript abundance, which subsequently remained at a relatively constant level through to harvest. Additionally, *CmADH4, CmADH5, CmADH7, CmADH9* and *CmADH11* were either not expressed or expressed at very low levels, and the transcript levels of *CmADH6* were always weakly maintained during fruit development.

### Effects of Hormones on *CmADH* Genes Expressions

**FIGURE 6:**
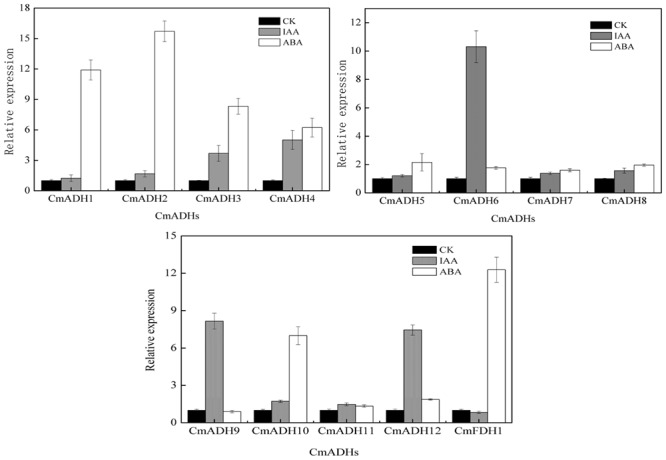
**The expression of *CmADHs* in melon fruit after treatments with IAA and ABA.** IAA and ABA (100 μM) treatments were given for 2 h as described in Section “Materials and Methods.” Expression analysis was carried out by real-time PCR. For each gene, the relative abundance of mRNA was normalized against the *18S* in the corresponding samples. The expression level of the genes in untreated melon fruit by IAA and ABA was set as “1.0.” Data represented the means ± SD (*n* = 3) of three biological samples. The experiments were carried out in triplicate.

The real-time PCR and semi-quantitative PCR results revealed that *CmADHs* were induced by IAA and ABA (though expression was lower in case of ABA), and showed different responses to IAA and ABA, except *CmADH11* (**Figure 6**; **Supplementary Figure S13**). The *CmADH1-4, CmADH10* and *CmFDH1* were strongly induced by ABA, and the relative expression varied between these genes. Moreover, *CmADH2* showed highest transcript levels. In contrast, IAA treatment strongly induced *CmADH6, CmADH9* and *CmADH12* expression, while other *CmADHs* were seemed to be insensitive to treatment with IAA and continued to be weakly expressed at a basal level.

**FIGURE 7:**
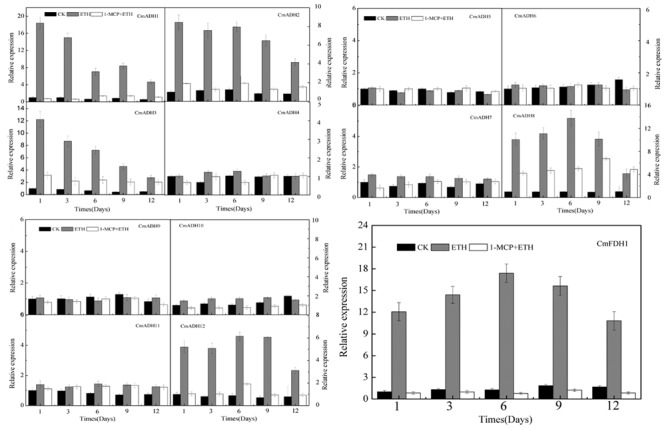
**The expression levels of *CmADHs* in melon fruit after treatments with ethylene and 1-MCP.** The transcript levels of *CmADHs* were measured by real-time PCR in melon fruit treated, and *18S* was used as internal control. The expression of the genes in untreated melon fruit after 1 day of storage was set to 1.0. Data represented the means ± SD (*n* = 3) of three biological samples. The experiments were carried out in triplicate.

Since oriental melons is a climacteric fruit which requires ethylene for initiation of ripening, we checked whether these *CmADHs* were regulated by ethylene. Transcript accumulation of different *CmADHs* in melon were investigated during the course of ethylene induced ripening of harvested melon for 12 days at 23°C. A basal level of expression of *CmADAHs* transcript was detected in all of 12 ADH genes and one FDH gene in ethylene untreated fruit on day 1. The transcript levels of *CmADH1-3, CmADH8, CmADH12* and *CmFDH1* shot up on day 1 in ethylene treated fruit, apart from *CmADH4-*7, *CmADH9* and *CmADH11* (**Figure 7**; **Supplementary Figure S14**). However, levels of *CmADH1, CmADH2* and *CmADH3* transcript gradually decreased from day 1 levels in subsequent days, but levels of *CmADH8, CmADH12 and CmFDH1* gradually increased from day 1 levels, and reduced from day 6 thereafter. Of the 12 ADH genes, *CmADH4-7, CmADH9* and *CmADH11* transcripts showed the lowest steady state levels in all of treatments during the course of melon ripening. Since these 6 *CmADHs* clustered into the medium-chain ADH subfamily, we checked the expression of *CmADH4-7, CmADH9* and *CmADH11* transcripts by semi-quantitative PCR at high annealing temperature using gene-specific primers to ensure that the transcript patterns were not due to cross hybridization. We found that these 6 *CmADHs* transcript levels were still lower and did not give clear results when 20 cycles of PCR were carried out as in case of other ADH genes. So for all semi-quantitative PCR experiments related to *CmADH4-7, CmADH9* and *CmADH11*, 35 cycles of PCR were carried out. Our results of semi-quantitative PCRs matched with real time qPCR analysis, and confirmed that all 12 ADHs and one FDH were expressed at different levels during ripening. The expressions of *CmADHs* were also studied in 1-MCP + ethylene treated fruits. All 12 ADHs and one FDH showed transcript accumulation in 1-MCP + ethylene treated fruits, but the levels were far lower compared to the levels in ethylene treated fruits except *CmADH4*-7, *CmADH9, CmADH10* and *CmADH11*, especially on day 1 post treatment (**Figure 7**; **Supplementary Figure S14**). Furthermore, *CmADH10* always showed stable and strong expression in all of treatments during the course of melon ripening. Therefore, these finding implied that *CmADH4*-7 and *CmADH9*-11 appeared to be not induced by ethylene. Since the patterns were same in real-time qPCR and semi-quantitative PCR experiments, further studies will be required.

## Discussion

Alcohol dehydrogenases, belonging to a group of dehydrogenase/reductases superfamily, are broadly distributed in all types of organisms and represent one of the most abundant classes of enzymes. More importantly, the ADH genes are involved in stress responses, elicitors and ABA (Matton et al., 1990; Kim et al., 2009; Strommer, 2011), and also closely associated with seedling, pollen development, root tissues, fruit ripening and aroma synthesis (Dolferus et al., 1994; Chung and Ferl, 1999; ManrÍquez et al., 2006; Singh et al., 2010; Kubienova et al., 2014). The ADH gene family has been investigated in a number of plant species, such as grape (Tesniere et al., 2004), Mango (Singh et al., 2010), tomato (Speirs et al., 1998), peaches (Zhang et al., 2010), apple (Defilippi et al., 2005a), soybean (Komatsu et al., 2011) and so on. The aim of the present study was to identify and analyze genes coding for the ADH enzyme in melon.

### Bioinformatic Analysis

In the melon genome database, we identified 13 ADH/ADH-like genes (including 12 ADHs and 1 FDH) coding for putative ADH enzymes, in which *CmADH1* and *CmADH2* have been cloned and characterized (ManrÍquez et al., 2006). Our research results also demonstrated that these melon ADH genes had very low sequence identity at protein level (ranging from 4.8 to 52.48%) and different intron-exon patterns at nucleotide level (**Figure 3**). Up to the present, research on ADH in plants have been focused on the medium- and short- chain ADH superfamilies (ManrÍquez et al., 2006; Kim et al., 2009; Singh et al., 2010; Iaria et al., 2012; Yamauchi et al., 2014; Chen et al., 2015). Furthermore, plant FDH, with hydroxylmethyl-glutathione or formaldehyde as preferred substrates, belonged to the medium-chain ADHs (Strommer, 2011), while it is weakly active or inactive on n-octanol or ethanol (Chase, 1999); Hence, in regard to substrate specifity, FDH was different from ADH1 in plant. In our study, *CmFDH1* clustered in medium-chain ADH superfamily, but the amino acid sequence of *CmFDH1* shared 28.33% identity with *CmADH1*. Based on the convergent evolution and structure of ADH gene-families in plant and animal, the plant ADH family was deemed to originate from a glutathione-dependent formaldehyde dehydrogenase gene (GSH-FDH) (Strommer, 2011). In maize, rice, pea and *Arabidopsis*, FDH genes were cloned and purified, but their functions in plants were not yet clear (Chase, 1999). As of present, there is still insufficient information on the function of FDHs in higher plants (Espunya et al., 2006; Strommer, 2011; Nian et al., 2013), hence it would be widely concerned by researchers in future.

The phylogenetic tree showed that ADH genes in melon clustered into 3 groups (**Figure 2**), indicating that ADH genes in melon exist in multiple isoforms. However, the phylogenetic tree obtained differed from previous analyses which grouped the plant ADH genes into two main classes (Chase, 1999; Duester et al., 1999; Thompson et al., 2007; Borràs et al., 2014), without the long-chain or Iron-activated ADH reported in plants. At the same time, we found that Cm*ADHs* sequences clustered close to that of other various species, suggesting that there are species- or lineage-specific ADH gene duplications (Komatsu et al., 2011) or a number of separate duplication events within melon which may be not homologous (Thompson et al., 2007). Additionally, these medium-chain *CmADHs*, divided into six subgroups (**Supplementary Figure S1** and **S4–S8**), also suggested that it seems possible to be different substrate specificities among melon medium-chain ADH genes and to be the same with that of vertebrates in which the medium-chain ADHs clustered in different subgroups (Duester et al., 1999). Hitherto, melon *CmADH1* (ManrÍquez et al., 2006), tomato *Le-ADH2* and *LeADH3* (Speirs et al., 1998), mango *MiADH1,2* (Singh et al., 2010), grape *Vv-ADH2* (Tesniere et al., 2004) and olive *OeADH* (Iaria et al., 2012), clustering a same class, belonged to the medium-chain zinc-binding type of ADHs (**Supplementary Figure S1**), and *in vitro*, the functional verification results revealed that this type of plant ADHs were implicated in plant development, fruit ripening and aroma production, and response to stresses.

Interestingly, contrary to medium-chain ADHs, *CmADH11* only retained a conserved functional protein domain, Zn2-binding region [Cys(102), Cys(105), Cys(108) and Cys(116)] (**Supplementary Figure S9**). Although *CmADH1, CmADH4* and *CmADH11* were grouped into a class in the phylogenetic tree (**Figure 2**), *CmADH11* showed a lower degree of homology to *CmADH1* and *CmADH4*, implying that *CmADH11* may be a member of zinc-medium-chain ADHs subfamily, and that these 3 *CmADHs* probably have similar functions, but more researches are required to examine this speculation. In addition, short-chain ADHs were found to be present in all living kingdoms. In tomato, a short-chain ADH, SlscADH1 protein contained motifs characteristic of classical short-chain dehydrogenases (Persson et al., 1991; Moummou et al., 2012). Nevertheless, in melon and ginseng, the protein sequence of short-chain ADH only encompassed some of these conserved regions, which were demonstrated to be related to substrate binding and active sites (ManrÍquez et al., 2006; Kim et al., 2009; Moummou et al., 2012). Furthermore, we found that the deduced protein of *CmADH12*, encoded by 635 amino acids, appeared to belong to long-chain ADH subfamily. In microorganisms, the sequences of the so-called long-chain or iron-dependent ADH genes were encoded by 600–750 amino acids, or up to over 900 amino acids (Williamson and Paquin, 1987; Goodlove et al., 1989; Chase, 1999). Whereas, *CmADH12* did not has the typical protein domains of long-chain or iron-dependent ADH proteins, possessed in corresponding ADHs of prokaryotes, human and other mammals (Inoue et al., 1989; Deng et al., 2002; Lyon et al., 2009; Elleuche et al., 2013, 2014). Interestingly, CmADH12 protein sequence exhibited two motifs typical of classical SDR-ADHs, namely an N-terminal coenzyme-binding pattern of typically GXXXGXG and an active-site pattern of YXXXK (**Supplementary Figure S11**); Simultaneously, this sequence possessed a NADPH-binding domain AXXGGTG that appears to be motifs typical of classical medium-chain ADHs by NCBI search, implying that CmADH12 may be consisted of a short-chain dehydrogenase protein and a medium-chain dehydrogenase. Up to now, for the long-chain ADH genes or iron-activated ADHs, no member of this type ADHs has been described in plant. Here, we reported a novel melon ADH, and the majority of predicted *CmADH12* in melon genome still awaits functional annotation. With the report of melon, *CmADH1* and *CmADH2*, belonging to the medium-chain ADHs and short-chain ADHs respectively (ManrÍquez et al., 2006), the identification of a long-chain ADH, *CmADH12*, will make a bridge to connect the gap in the distribution of medium- and short-chain ADHs, and will represent significant improvements on the understanding of the evolution of ADH in higher plants.

### Expression Analysis in Different Tissue

In plants, multiple homologous ADH genes, thought to have distinct roles, have been implicated in the response to biotic and abiotic stresses, particularly, in the tissues development under stresses (Abe et al., 2003; Delessert et al., 2004; Kumar et al., 2010; Frazier et al., 2011; Komatsu et al., 2011; Qi et al., 2012). At the same time, many previous studies indicated that plant ADHs have been involved in seedling, pollen development and fruit development (Kim et al., 2010; Thompson et al., 2010), suggesting that ADH genes play a important role in progress of the vegetative and reproductive organs development. In melon, *CmADHs* also showed different expression patterns in tissues and fruits during different development stages (**Figures 4** and **5**; **Supplementary Figure S12**). *CmADH1* and *CmADH2* were weakly expressed or not expressed in leaf, stem, seed, root, and flower of Cantaloupe (ManrÍquez et al., 2006), without the same as our results (**Figure 4**; **Supplementary Figure S12**). Hence, we speculated that it seems to be related to sample type or other, which are worthy of further study. In mango, all of three *MiADHs* were weakly expressed in flower, leaf, and stem, with different transcript levels between *MiADH*s (Singh et al., 2010). In *petunia hybrid*, three functional ADHs had different expressions in immature pollen grains, anther, stigma, petal, ovary and hypoxic root (Garabagi and Strommer, 2004); Moreover, particularly in the nectary, ADH3 was remarkably expressed, suggesting the association of ADH genes with production of aromatic compounds in the nectary of *petunia hybrid* (Garabagi et al., 2005). In the present study, *CmADH1, CmADH2, CmADH10* and *CmFDH1* were strongly expressed in petal, with a higher corresponding enzymatic activity in pistillate and staminate flower petal respectively (**Supplementary Figure S15**). Therefore, we infered that these four *CmADHs* participated in the flowers development and the production of volatile substances in melon flowers, and based on these findings, the previously assumed functional redundancy of these genes in the ADH network needs to be questioned (Strommer, 2011).

In addition, plant ADH genes were also implicated in fruit tissue development and fruit ripening, and were closely related to aroma volatile production. For example, mango flesh undergone softening during ripening, and *MiADH1* and *MiADH2* transcripts particularly accumulated at the onset of ripening in mango fruit, and gradually reduced thereafter; whereas, *MiADH3* accumulated during early development of fruit (Singh et al., 2010). In tamato, *SlscADH1*, a fruit-ripening-associated short-chain ADH, has been related to the formation of aroma volatiles (Gaut et al., 1999). In the present study, the ADH enzyme activity increased at first and then decreased, and had a peak at 36 days after the anthesis during fruit development (**Supplementary Figure S16**). Nevertheless, only *CmADH2* and *CmADH10* were strongly expressed during the progress of fruit development except 15 days after the anthesis, suggesting that *CmADH2* and *CmADH10* may participate in fruit development of the whole development period of melon. In olea europaea, *OeADH* transcript guadually rose along with olea fruit development and peaked at fruit ripening, and the change of *OeADH* expression was coherent with the olea fruit growth curve (Iaria et al., 2012), and the similar pattern of *ADH* gene expression was found in apricot fruits (Gonzalez-Agüero et al., 2009). Additionally, in Cantaloupe, the transcript levels of *CmADH1* and *CmADH2* gradually increased at first and then decreased from 32 to 42 days with a peak at 39 days (ManrÍquez et al., 2006). However, our data showed *CmADH1* was strongly expressed at the late stage of development; *CmADH2* transcripts always maintained higher levels during fruit development (**Supplementary Figure S12**). In addition, *VvADH1* and *VvADH3* in grape were temporarily expressed in young and developing berry, whereas *VvAdh2* transcript was strongly accumulated at later developmental stages, from the onset of ripening (Tesnière and Verriès, 2000). In melon, *CmADH1, CmADH3, CmADH8* and *CmFDH1* were highly expressed in fruit at 1 day after anthesis, suggesting that these 4 ADH genes likely participate in the ovary development of melon fruit. Of three functional petunia ADHs, *PhADH2* and *PhADH3* were expressed in ovary (Garabagi et al., 2005). As for melons, little information was available on the role of ADH in relation to the ovary development of fruit. Furthermore, expression analysis also indicated that *CmADH8* and *CmFDH1* were prominently expressed later in fruit development. However, little information was available on the role of another medium-chain ADH in relation to melon fruit development and ripening. These findings implied that *CmADH8* and *CmFDH1* might also be involved in fruit development or in other unknown functions at different developmental stages, and also suggested that medium-chain *CmADHs* might have evolved individual unique functions in fruit development, but it appears that a complex network, consisted of the members of ADH superfamily, plays a important role to modulate the fruit development and ripening. There is much to learn about medium-chain ADHs in melon.

There were significant expression differences between *CmADH11* and other medium-chain *CmADHs* in different tissues and during development and ripening. These observed differences could partly be explained by the amino acids differences at the Zn1-binding sequences and the coenzyme binding position (**Supplementary Figure S9**). Based on our analysis of medium-chan *CmADHs, CmADH11* had little key residues, and had Phe, Ala, and Tyr instead of three Gly in the coenzyme binding domain GXG(X)_2_G of all functional medium-chain ADHs. Furthermore, some substituted residues in soybean ADH2 protein might retain the function of amino acids, and it is not clear in other substitutions (Komatsu et al., 2011). Accordingly, these findings implied difference in enzymatic properties between *CmADH11* and *CmADH1* and other plant ADHs. On the basis of the above results, it appears that *CmADH11* is a pseudogene. But we used the expressed sequence tag (EST) database in NCBI as a source of mRNA sequence bioinformatics for *CmADH11*, and we found that *CmADH11* showed the highest homology with a *Cucumis melo* cDNA clone (GenBank no.JG552191.1) (89% identity), obtained from callus, from EST database of melon (CM-DEa library). Hence, it appears that *CmADH11* is specifically expressed in callus. Further research is required to confirm this observation.

### Induced Expression Analysis

We also studied the effect of ABA and IAA on *CmADHs* expression. ABA is known to be extensively involved in plant developmental processes and the plant’s response to abiotic stresses, and can regulate the expression of relevant genes to increase plant adaptability and to regulate tissues development (Skriver and Mundy, 1990; Matsukura et al., 2010; Su et al., 2013). ABA has been shown to induce ADH gene expression in *Arabidopsis* (de Bruxelles et al., 1996; Su et al., 2013), barley (Macnicol and Jacobsen, 2001) and mango (Singh et al., 2010); In particular, maize (Hwang and Vantoai, 1991) and lettuce (Kato-Noguchi, 2000) ADH genes were strongly induced under abiotic stresses. Purportedly, ABA-mediated plant responses to drought stress might be related to the regulation of relevant genes by key regulator in ABA signaling, including MYB, MYC, NAC, PP2Cs, DREB family and so on (Abe et al., 2003; Fujita et al., 2011; Su et al., 2013). However, to the best of our information, there were little reports on effect of ABA or IAA on ripening specific ADHs in melon. Promoter analysis of the 12 melon ADH genes and one FDH gene suggested the presence of ABA responsive ABRE motif in the promoter of *CmFDH1, CmADH2, CmADH3* and *CmADH10* (Su et al., 2013) (Supplementary Table S1). Additionally, the promoter of mango ADHs has the ABA responsive ABRE motif (Gomez-Porras et al., 2007; Singh et al., 2010), while *MiADH1* was induced by ABA, and *MiADH2* was repressed. In the present study, ABA treatment boosted the expressions of *CmADH1, CmADH2, CmADH3, CmADH4, CmADH10* and *CmFDH1*, but ABA did not obviously inhibit other ADHs transcripts (**Figure 6**; **Supplementary Figure S13**). Therefore, the ABA-induced these *CmADHs* expression observed in present study may be related to the upstream key transcription factors. However, this speculation has to be demonstrated by further cloning and function analyses of the promoter of *CmADHs*. Furthermore, co-treatment with ABA and ethylene enhanced *MiADH1* expression which was higher than those by either of the hormone alone, and ethylene could relieve the inhibition of ABA on *MiADH2* transcript, suggesting that there were likely mutual promotion between ABA and ethylene, or that ethylene was in some manner able to repress ABA action (Singh et al., 2010). All of these derived results also implied that it would be important to investigate whether ABA triggers different hormonal signal pahways, particularly the possible crosstalk between ABA and ethylene, because ABA and ethylene are key hormones in fruit ripening and senescence, as hormone crosstalk has been shown to be important for biotic and abiotic stresses (Bari and Jones, 2009; Su et al., 2013). More further work is needed to investigate the crosstalk in response to fruit ripening. Auxin (IAA) also plays a role in fruit development and ripening (Singh et al., 2010), and there was an active crosstalk between IAA and ethylene during fruit ripening (Trainotti et al., 2007). However, in the case of melon, there were not reports about the inducing of ripening in mature fruit by IAA as done by ethylene. In tamato, the mutual effect between IAA and ethylene boosted tomato fruit development at early developmental stages (Balbi and Lomax, 2003). Three mango ADH genes expressions were not obviously induced by IAA treatment, and IAA did not induced the mango fruit ripening, moreover there are not IAA responsive TGA motif in promoter in mango ADHs (Singh et al., 2010). *CmADH6, CmADH9* and *CmADH12* promoters contained IAA responsive TGA motif, and IAA treatment induced these three ADHs transcripts (**Figure 6**; **Supplementary Figure S13**). Other ADH genes were expressed at basal level or not expressed, but did not appear to be significantly regulated by IAA, which were consistent with observations in mango (Singh et al., 2010).

Our results also indicated that 12 *CmADHs* and 1 *CmFDH* showed different patterns of transcripts during ethylene induced ripening (**Figure 7**; **Supplementary Figure S14**), which could be explained by the presence of ethylene responsive ERE motifs in *CmADH1, CmADH2, CmADH3, CmADH8, CmADH12* and *CmFDH1* (Tesniere et al., 2004; Jin et al., 2014) (Supplementary Table S1). According to previous reports, there are ethylene responsive ERE motifs in *Arabidopsis AtADH1* (Peng et al., 2001), mango *MiADH1* (Singh et al., 2010) and tomato *LeADH2* (Speirs et al., 1998) promoters, and these ADH genes were responsive to ethylene. So, in view of melon ADH genes responses to ethylene, *CmADH1, CmADH2, CmADH3, CmADH8, CmADH12* and *CmFDH1* were classed into ethylene-sensitive group, and other *CmADHs* clustered ethylene-insensitive group. Furthermore, Manråquez et al.(2006) also found that ethylene increased the levels of *CmADH1* and *CmADH2* transcripts in Cantaloupe during fruit ripening, while 1-MCP treatment inhibited the inducing effect of ethylene to *CmADH1* and *CmADH2*. This was similar to the ripening related expression observed in mango ADHs (Singh et al., 2010), tomato *LeADH2* (climacteric fruit) (Defilippi et al., 2005b; Barakat et al., 2009) and *VvADH2* in grape (non-climacteric fruit) (Tesniere et al., 2004). Simultaneously, ethylene treatment enhanced the activity of ADH enzyme in mango and ethylene production and accelerated mango fruit ripening and senescence, but the increase effect of ethylene was weakened by 1-MCP (Singh et al., 2010). Whereas, in tomato, ADH enzymatic activity increased during fruit ripening, and *LeADH2* was involved in fruit ripening and aroma compounds synthesis, but was not regulated by ethylene (Speirs et al., 1998, 2002); Ethylene induced the increases of ADH enzymatic activity and *VvADH2* transcript in grape (a climacteric fruit) during fruit ripening (Tesniere et al., 2004), but did not clearly enhanced the activity of ADH enzyme in ACC oxidase antisense transgenic apple, without influence on the esters production in this kind of apple (Defilippi et al., 2005a). These findings showed that the ADH genes superfamily included ethylene non-dependent ADH gene members in climacteric kinds of fruit and ethylene dependent ADH gene members in non-climacteric kinds of fruit. In this paper, the changes of ADH enzyme activity in fruit treated by ethylene and 1-MCP (**Supplementary Figure S17**) and *CmADH10* expression indicated that *CmADH10* may be a non-dependent ADH gene member (**Figure 7**; **Supplementary Figure S14**). Whereas, more experimental works are needed to further elucidate the function of *CmADH10* in aroma compounds synthesis and during fruit ripening and senescence.

Additionally, we also identified several hormone-responsive *cis*-regulatory elements in the *CmADHs* promoter region, such as GARE, TATC-box, P-box (gibberellin), TCA-element (SA), CCAAT-box, TGACG-motif and CGTCA-motif (MeJA) (Barakat et al., 2009; Kim et al., 2010; Jin et al., 2014) (Supplementary Table S3). Several previous studies showed a complex interplay of hormones was known to affect fruit development and ripening, with auxin and GA being important during fruit expansion and ABA and ethylene being important for ripening (Srivastava and Handa, 2005; Singh et al., 2010). In addition, oriental melon (*Cucumis melo* var. *makuwa* Makino) has distinct ripening patterns within the fruit (Jin et al., 2014). Therefore, based on our derived results in this study, we speculated that the different regulation of melon fruit ripening by ABA, IAA and ethylene might selectively affect the expression of one- or multi- ADHs, which prompted *CmADHs* to regulate fruit ripening and senescence. Maybe, it will be possible to retain a crosstalk between ABA/IAA/JA/GA and ethylene to regulate the ADH genes network involved in fruit development and ripening, as hormone crosstalk has been shown to be important for biotic and abiotic stresses (Bari and Jones, 2009; Su et al., 2013). Further research is required to confirm this hypothesis.

## Conclusion

We identified 13 CmADHs (including 12 ADHs and 1 FDH) in melon genome, exhibiting lower identity in protein level with each other, and they were expressed differentially in different vegetative tissues and during fruit development and ripening. Phylogenetic analysis indicated that they belonged to three different ADH subfamilies and the medium-chain ADHs were clustered into six subgroups. On the transcript level, differential *CmADHs* expression suggested tight adaptation of the fruit to the developmental events. Promoter sequence analysis, intron-exon structure and the different responses of these ADH genes to ABA, IAA and ethylene implied that ADH genes had different functions or distinct metabolic roles in melon fruit development and ripening, and the different ADH genes expression at transcript induced by ethylene indicated that *CmADH10* was an ethylene non-dependent ADH gene member in *CmADH* genes. Further and detailed research efforts should be made to find the detailed function of these ADH proteins in addition to *CmADH1* and *CmADH2* during melon fruit development and ripening. Therefore, selective silencing of the *CmADH* genes and more studies related to biochemical characterization of these *CmADHs* will be needed.

## Author Contributions

Conceived and designed the experiments: YJ, HQ, CZ; Performed the experiments: YJ, CZ, WL, HC, SC; Analyzed the data: YJ, CZ, HQ, HC; Contributed reagents/materials/analysis tools: YJ, CZ, YT; Wrote the paper: YJ, CZ, HQ.

## Conflict of Interest Statement

The authors declare that the research was conducted in the absence of any commercial or financial relationships that could be construed as a potential conflict of interest.

## Footnotes

1 http://melonomics.net

2 http://megasoftware.net

3 http://frodo.wi.mit.edu/

4 http://www.ncbi.nlm.nih.gov/Structure/cdd/wrpsb.cgi

## Supplementary Material

FIGURE S1 **Phylogenetic tree of melon medium-chain ADH proteins.** The amino acid sequences were aligned by the Clustal Omega program, and the neighborjoining tree was drawn with TreeView. The corresponding GenBank and the melon genome (https://melonomics.net/) were noted in the phylogenetic tree and the accession number in the melon genome were *CmADH1* (MELO3C023685P4), *CmADH3* (MELO3C026552P1), *CmADH4* (MELO3C027151P1), *CmADH5* (MELO3C00579 2P1), *CmADH6* (MELO3C026553P1), *CmADH7* (MELO3C002189P1), *CmADH8* (ME LO3C003251P1), *CmADH9* (MELO3C011043P1), *CmADH10* (MELO3C026554P2), *CmADH11* (MELO3C023687P1), and *CmFDH1* (MELO3C022399P1). The number for each interior branch was the percentage of bootstraps value (1000 replicates). Black circle denoted 13 CmADHs. ADHs from other plants in our paper are in Supplementary Table S3.

FIGURE S2 **Amino acid sequence alignment of melon *CmADH1* (MELO3C023685P4) and *CmADH4* (MELO3C027151 P1) with closely related sequences of other plants.** GenBank accession numbers are in Supplementary Table S3.

FIGURE S3 **Amino acid sequence alignment of melon *CmFDH1* (MELO3C022399P1) with closely related sequences of other plants.** GenBank accession numbers are in Supplementary Table S3.

FIGURE S4 **Amino acid sequence alignment of melon *CmADH3* (MELO3C026552P1), *CmADH6* (MELO3C026553P1) and *CmADH10* (MELO3C026554P2) with closely related sequences of other plants.** GenBank accession numbers are in Supplementary Table S3.

FIGURE S5 **Amino acid sequence alignment of melon *CmADH5* (MELO3C005 792P1) with closely related sequences of other plants.** GenBank accession numbers are in Supplementary Table S3.

FIGURE S6 **Amino acid sequence alignment of melon *CmADH7* (MELO3C002 189P1) with closely related sequences of other plants.** GenBank accession numbers are in Supplementary Table S3.

FIGURE S7 **Amino acid sequence alignment of melon *CmADH8* (MELO3C003 251P1) with closely related sequences of other plants.** GenBank accession numbers are in Supplementary Table S3.

FIGURE S8 **Amino acid sequence alignment of melon *CmADH9* (MELO3C011043 P1) with closely related sequences of other plants.** GenBank accession numbers are in Supplementary Table S3.

FIGURE S9 **Amino acid sequence alignment of melon *CmADH11* (MELO3C023 687P1) with closely related sequences of other plants.** GenBank accession numbers are in Supplementary Table S3.

FIGURE S10 **Amino acid sequence alignment of melon *CmADH2* (MELO3C014 897P1) with closely related sequences of other plants.** GenBank accession numbers are in Supplementary Table S3.

FIGURE S11 **Amino acid sequence alignment of melon *CmADH12* (MELO3C019 503P1) with closely related sequences of other plants.** GenBank accession numbers are in Supplementary Table S3.

FIGURE S12 **Transcript abundance of *CmADHs* in vegetative tissues and developing stages of melon fruit (1–48).** R, root; ML, mature leaf; DL, developing leaf; YS, young stem; PF, pistillate flower petal; SF, staminate flower petal. RNA isolated from the samples. Expression analysis was carried out by semi-quantitative PCR using *18S* as an internal control.

FIGURE S13 **mRNA abundance of *CmADHs* in mature green fruit after treatment with ABA and IAA.** IAA (Auxin) and ABA (100μM) treatments were given for 2 h as described in Section “Materials and Methods.” Expression analysis was carried out by semi-quantitative PCR as described in section of Hormone treatments using actin as *18S* internal control.

FIGURE S14 ***CmADHs* accumulation during different stages of ethylene and 1-MCP treatment induced ripening of melon by semi-quantitative PCR.** 1–12 indicate days after ethylene and 1-MCP treatment. The accumulation level of the genes in untreated melon fruit was control. *18S* was used as an internal control.

FIGURE S15 **Alcohol dehydrogenases activity in different melon organs.** R, root; ML, mature leaf; DL, developing leaf; YS, young stem; PF, pistillate flower petal; SF, staminate flower petal.

FIGURE S16 **Alcohol dehydrogenases activity in fruits during melon development.** 1, 5, 10, 15, 20, 25, 30, 33, 36, 39, 42, 45, and 48 indicate days after anthesis.

FIGURE S17 **Alcohol dehydrogenases activity in fruits during different stages of ethylene and 1-MCP treatment induced ripening of melon.** 1, 3, 6, 9, and 12 indicate days after ethylene and 1-MCP treatment. All the treatments have been described in section of Hormone treatments.

## References

[B1] Abe H. Urao T. Ito T. Seki M. Shinozaki K. Yamaguchi-Shinozaki K. (2003). *Arabidopsis* AtMYC2 (bHLH) and AtMYB2 (MYB) function as transcriptional activators in abscisic acid signaling. *Plant Cell* 15 63–78. 10.1105/tpc.006130 12509522 PMC143451

[B2] Alka K. Windle H. J. Cornally D. Ryan B. J. Henehan G. T. M. (2013). A short chain NAD(H)-dependent alcohol dehydrogenase (HpSCADH) from *Helicobacter pylori*: a role in growth under neutral and acidic conditions. *Int. J. Biochem. Cell Biol.* 45 1347–1355. 10.1016/j.biocel.2013.04.006 23583739

[B3] Balbi V. Lomax T. L. (2003). Regulation of early tomato fruit development by the diageotropica gene. *Plant Physiol.* 131 186–197. 10.1104/pp.010132 12529527 PMC166799

[B4] Barakat A. Bagniewska-Zadworna A. Choi A. Plakkat U. DiLoreto D. Yellanki P. (2009). The cinnamyl alcohol dehydrogenase gene family in *Populus*: phylogeny, organization, and expression. *BMC Plant Biol.* 9:26 10.1186/1471-2229-9-26 PMC2662859 19267902

[B5] Bari R. Jones J. D. (2009). Role of plant hormones in plant defence responses. *Plant Mol. Biol.* 69 473–488. 10.1007/s11103-008-9435-0 19083153

[B6] Borràs E. Albalat R. Duester G. Parés X. Farrés J. (2014). The *Xenopus* alcohol dehydrogenase gene family: characterization and comparative analysis incorporating amphibian and reptilian genomes. *BMC Genomics* 15:216 10.1186/1471-2164-15-216 PMC4028059 24649825

[B7] Bukh C. Nord-Larsen P. H. Rasmussen S. K. (2012). Phylogeny and structure of the cinnamyl alcohol dehydrogenase gene family in *Brachypodium distachyon*. *J. Exp. Bot.* 63 6223–6236. 10.1093/jxb/ers275 23028019 PMC3481213

[B8] Çelik A. Aktas F. A. (2013). A new NADH-dependent, zinc containing alcohol dehydrogenase from *Bacillus thuringiensis* serovar israelensis involved in oxidations of short to medium chain primary alcohols. *J. Mol. Catal. B Enzym.* 89 114–121. 10.1016/j.molcatb.2013.01.005

[B9] Charlesworth D. Liu F. L. Zhang L. (1998). The evolution of the alcohol dehydrogenase gene family by loss of introns in plants of the genus *Leavenworthia* (Brassicaceae). *Mol. Biol. Evol.* 15 552–559. 10.1093/oxfordjournals.molbev.a025955 9580984

[B10] Chase T. (1999). Alcohol dehydrogenases: identification and names for gene families. *Plant Mol. Biol. Rep.* 17 333–350. 10.1023/A:1007620627083

[B11] Chen F. F. Wang P. An Y. A. Huang J. Q. Xu Y. W. (2015). Structural insight into the conformational change of alcohol dehydrogenase from *Arabidopsis thaliana* L. during coenzyme binding. *Biochimie* 108 33–39. 10.1016/j.biochi.2014.10.023 25447145

[B12] Cheng F. F. Hu T. An Y. Huang J. Q. Xu Y. W. (2013). Purification and enzymatic characterization of alcohol dehydrogenase from *Arabidopsis thaliana*. *Protein Expr. Purif.* 90 74–77. 10.1016/j.pep.2013.05.004 23707506

[B13] Cheng H. Li L. L. Xu F. Cheng S. Y. Cao F. L. Wang Y. (2013). Expression patterns of a cinnamyl alcohol dehydrogenase gene involved in lignin biosynthesis and environmental stress in *Ginkgo biloba*. *Mol. Biol. Rep.* 40 707–721. 10.1007/s11033-012-2111-0 23143181

[B14] Chung H. J. Ferl R. J. (1999). *Arabidopsis* alcohol dehydrogenase expression in both shoots and roots is conditioned by root growth environment. *Plant Physiol.* 121 429–436. 10.1104/pp.121.2.429 10517834 PMC59405

[B15] de Bruxelles G. L. Peacock W. J. Dennis E. S. Dolferus R. (1996). Abscisic acid induces the alcohol dehydrogenase gene in *Arabidopsis*. *Plant Physiol.* 111 381–391. 10.1104/pp.111.2.381 8787023 PMC157847

[B16] Defilippi B. G. Dandekar A. M. Kader A. A. (2005a). Relationship of ethylene biosynthesis tovolatile production, related enzymesand precursor availability in apple peel and flesh tissues. *J. Agric. Food Chem.* 53 3133–3141. 10.1021/jf047892x 15826070

[B17] Defilippi B. G. Kader A. A. Dandekar A. M. (2005b). Apple aroma: alcohol acyltransferase, a rate limiting step for ester biosynthesis, is regulated by ethylene. *Plant Sci.* 168 199–210. 10.1016/j.plantsci.2004.12.018

[B18] Delessert C. Wilson I. W. Van Der Straeten D. Dennis E. S. Dolferus R. (2004). Spatial and temporal analysis of the local response to wounding in *Arabidopsis* leaves. *Plant Mol. Biol.* 55 165–181. 10.1007/s11103-004-0112-7 15604673

[B19] Deng Y. Wang Z. Gu S. Ji C. Ying K. Xie Y. (2002). Cloning and characterization of a novel human alcohol dehydrogenase gene (ADHFe1). *DNA Seq.* 13 301–306. 10.1080/1042517021000011636 12592711

[B20] Dolferus R. Jacobs M. Peacock W. J. Dennis E. S. (1994). Differential interactions of promoter elements in stress responses of the *Arabidopsis* Adh gene. *Plant Physiol.* 105 1075–1087. 10.1104/pp.105.4.1075 7972489 PMC159435

[B21] Dolferus R. Osterman J. Peacock W. J. Dennis E. S. (1997). Cloning of the *Arabidopsis* and rice class III Adh genes: implications for the origin of plant ADH enzymes. *BMC Genet.* 146:1131–1141. 10.1093/genetics/146.3.1131 PMC1208041 9215914

[B22] Duester G. Farrés J. Felder M. R. Holmes R. S. Höög J. O. Parés X. (1999). Recommended nomenclature for the vertebrate alcohol dehydrogenase gene family. *Biochem. Pharmacol.* 58 389–395. 10.1016/S0006-2952(99)00065-9 10424757

[B23] Elleuche S. Fodor K. Klippel B. Heyde A. Wilmanns M. Antranikian G. (2013). Structural and biochemical characterisation of a NAD+-dependent alcohol dehydrogenase from *Oenococcus oeni* as a new model molecule for industrial biotechnology applications. *Appl. Microbiol. Biotechnol.* 97 8963–8975. 10.1007/s00253-013-4725-0 23385476

[B24] Elleuche S. Fodor K. von der Heyde A. Klippel B. Wilmanns M. Antranikian G. (2014). Group III alcohol dehydrogenase from *Pectobacterium atrosepticum*: insights into enzymatic activity and organization of the metal ion-containing region. *Appl. Microbiol. Biotechnol.* 98 4041–4051. 10.1007/s00253-013-5374-z 24265029

[B25] Espunya M. C. De Michele R. Gomez-Cadenas A. Martinez M. C. (2012). S-nitrosoglutathione is a component of wound- and salicylic acid-induced systemic responses in *Arabidopsis thaliana*. *J. Exp. Bot.* 63 3219–3227. 10.1093/jxb/ers043 22371078 PMC3350931

[B26] Espunya M. C. Díaz M. Moreno-Romero J. Martínez M. C. (2006). Modification of intracellular levels of glutathione-dependent formaldehyde dehydrogenase alters glutathione homeostasis and root development. *Plant Cell Environ.* 29 1002–1011. 10.1111/j.1365-3040.2006.01497.x 17087482

[B27] Feechan A. Kwon E. Yun B. W. Wang Y. Pallas J. A. Loake G. J. (2005). A central role for S-nitrosothiols in plant disease resistance. *Proc. Natl. Acad. Sci. U.S.A.* 102 8054–8059. 10.1073/pnas.0501456102 15911759 PMC1142375

[B28] Filling C. Berndt K. D. Benach J. Knapp S. Prozorovski T. Nordling E. (2002). Critical residues for structure and catalysis in short-chain dehydrogenases/reductases. *J. Biol. Chem.* 277 25677–25684. 10.1074/jbc.M202160200 11976334

[B29] Frazier T. P. Sun G. Burklew C. E. Zhang B. (2011). Salt and drought stresses induce the aberrant expression of microRNA genes in tobacco. *Mol. Biotechnol.* 49 159–165. 10.1007/s12033-011-9387-5 21359858

[B30] Fujita Y. Fujita M. Shinozaki K. Yamaguchi-Shinozaki K. (2011). ABA-mediated transcriptional regulation in response to osmotic stress in plants. *J. Plant Res.* 124 1–17. 10.1007/s10265-011-0412-3 21416314

[B31] Garabagi F. Duns G. Strommer J. (2005). Selective recruitment of Adh genes for distinct enzymatic functions in Petunia hybrid. *Plant Mol. Biol.* 58 283–294. 10.1007/s11103-005-3545-8 16027979

[B32] Garabagi F. Strommer J. (2004). Distinct genes produce the alcohol dehydrogenases of pollen and maternal tissues in *Petunia hybrida*. *Biochem. Genet.* 42 199–208. 10.1023/B:BIGI.0000026634.69911.2e 15260144

[B33] Garcia-Mas J. Benjak A. Sanseverino W. Bourgeois M. Mir G. González V. M. (2012). The genome of melon (*Cucumis melo* L.). *Proc. Natl. Acad. Sci. U.S.A.* 109 11872–11877. 10.1073/pnas.1205415109 22753475 PMC3406823

[B34] Gaut B. S. Peek A. S. Morton B. R. Clegg M. T. (1999). Patterns of genetic diversification within the Adh gene family in the grasses (Poaceae). *Mol. Biol. Evol.* 16 1086–1097. 10.1093/oxfordjournals.molbev.a026198 10474904

[B35] Gomez-Porras J. L. Riano-Pachon D. M. Dreyer I. Mayer J. E. (2007). Mueller-Roeber B. Genome-wide analysis of ABA-responsive elements ABRE and CE3. *BMC Genomics* 8:260 10.1186/1471-2164-8-260 PMC2000901 17672917

[B36] Gonzalez-Agüero M. Troncoso S. Gudenschwager O. Campos-Vargas R. Moya-Leon M. A. Defilippi B. G. (2009). Differential expression levels of aroma-related genes during ripening of apricot (*Prunusarmeniaca* L.). *Plant Physiol. Biochem.* 47 435–440. 10.1016/j.plaphy.2009.01.002 19233665

[B37] Goodlove P. E. Cunningham P. R. Parker J. Clark D. P. (1989). Cloning and sequence analysis of the fermentative alcohol-dehydrogenase-encoding gene of *Escherichia coli*. *Gene* 85 209–214. 10.1016/0378-1119(89)90483-6 2695398

[B38] Höög J. O. Strömberg P. Hedberg J. J. Griffiths W. J. (2003). The mammalian alcohol dehydrogenases interact in several metabolic pathways. *Chem. Biol. Interact.* 144 175–181. 12604202 10.1016/s0009-2797(02)00225-9

[B39] Hwang S. Y. Vantoai T. T. (1991). Abscisic acid induces anaerobiosis tolerance in corn. *Plant Physiol.* 97 593–597. 10.1104/pp.97.2.593 16668440 PMC1081048

[B40] Iaria D. L. Bruno L. Macchione B. Tagarelli A. Sindona G. Giannino D. (2012). The aroma biogenesis-related olea europaea alcohol dehydrogenase gene is developmentally regulated in the fruits of two *O. europaea* L. cultivars. *Food Res. Int.* 49 720–727. 10.1016/j.foodres.2012.09.004

[B41] Inoue T. Sunagawa M. Mori A. Imai M. Fukuda M. Takagi M. (1989). Cloning and sequencing of the gene encoding the 72-kilodalton dehydrogenase subunit of the alcohol dehydrogenase from *Acetobacter aceti*. *J. Bacteriol.* 171 3115–3122. 2722742 10.1128/jb.171.6.3115-3122.1989 PMC210023

[B42] Jin Y. Z. Zhang C. Liu W. Qi H. Y. Chen H. Cao S. X. (2014). The cinnamyl alcohol dehydrogenase gene family in melon (*Cucumis melo* L.): bioinformatic analysis and expression patterns. *PLoS ONE* 9:e101730 10.1371/journal.pone.0101730 PMC4096510 25019207

[B43] Joërnvall H. Hoëoëg J. O. Persson B. (1999). SDR and MDR: completed genome sequences show these protein families to be large, of old origin, and of complex nature. *FEBS Lett.* 445 261–264. 10.1016/S0014-5793(99)00130-1 10094468

[B44] Jönvall H. Hedlund J. Bergman T. Kallberg Y. Cederlund E. Persson B. (2013). Origin and evolution of medium chain alcohol dehydrogenases. *Chem. Biol. Interact.* 202 91–96. 10.1016/j.cbi.2012.11.008 23200944

[B45] Jönvall H. Hedlund J. Bergman T. Oppermann U. Persson B. (2010). Superfamilies SDR and MDR: from early ancestry to present forms. Emergence of three lines, a Zn-metalloenzyme, and distinct variabilities. *Biochem. Biophys. Res. Commun.* 396 125–130. 10.1016/j.bbrc.2010.03.094 20494124

[B46] Kato-Noguchi H. (2000). Abscisic acid and hypoxic induction of anoxia tolerance in roots of lettuce seedlings. *J. Exp. Bot.* 51 1939–1944. 10.1093/jexbot/51.352.1939 11113172

[B47] Khan A. J. Husain Q. Choudhuri G. Parmar D. (2010). Association of polymorphism in alcohol dehydrogenase and interaction with other genetic risk factors with alcoholic liver cirrhosis. *Drug Alcohol Depend.* 109 190–197. 10.1016/j.drugalcdep.2010.01.010 20171022

[B48] Kim Y. H. Bae J. M. Huh G. H. (2010). Transcriptional regulation of the cinnamyl alcohol dehydrogenase gene from sweetpotato in response to plant developmental stage and environmental stress. *Plant Cell Rep.* 29 779–791. 10.1007/s00299-010-0864-2 20454964 PMC2886125

[B49] Kim Y. J. Shim J. S. Lee J. H. Jung S. Y. Sun H. In J. G. (2009). Isolation and characterization of a novel short-chain alcohol dehydrogenase gene from *Panax ginseng*. *BMB Rep.* 42 673–678. 10.5483/BMBRep.2009.42.10.673 19874713

[B50] Komatsu S. Thibaut D. Hiraga S. Kato M. Chiba M. Hashiguchi A. (2011). Characterization of a novel flooding stress-responsive alcohol dehydrogenase expressed in soybean roots. *Plant Mol. Biol.* 7 309–322. 10.1007/s11103-011-9812-y 21811849

[B51] Koutsompogeras P. Kyriacou A. Zabetakis I. (2010). Characterization of NAD-dependent alcohol dehydrogenase enzymes of strawberry’s achenes (*Fragaria x ananassa* cv. Elsanta) and comparison with respective enzymes from *Methylobacterium extorquens*. *Food Sci. Technol.* 43 828–835.

[B52] Kubienova L. Ticha T. Jahnova J. Luhova L. Mieslerova B. Petrivalsky M. (2014). Effect of abiotic stress stimuli on S-nitrosoglutathione reductase in plants. *Planta* 239 139–146. 10.1007/s00425-013-1970-5 24104214

[B53] Kumar M. S. Hema R. Suryachandra T. R. Ramegowda H. V. Gopalakrishna R. Rama N. (2010). Functional characterization of three water deficit stress-induced genes in tobacco and *Arabidopsis*: an approach based on gene down regulation. *Plant Physiol. Biochem.* 48 35–44. 10.1016/j.plaphy.2009.09.005 19811926

[B54] Kumar S. Sandell L. L. Trainor P. A. Koentgend F. Duestera G. (2012). Alcohol and aldehyde dehydrogenases: retinoid metabolic effects in mouse knockout models. *Biochim. Biophys. Acta* 1821 198–205. 10.1016/j.bbalip.2011.04.004 21515404 PMC3161159

[B55] Lee U. Wie C. Fernandez B. O. Feelisch M. Vierling E. (2008). Modulation of nitrosative stress by S-nitrosoglutathione reductase is critical for thermotolerance and plant growth in *Arabidopsis*. *Plant Cell* 20 786–802. 10.1105/tpc.107.052647 18326829 PMC2329944

[B56] Lyon R. C. Johnston S. M. Panopoulos A. Alzeer S. McGarvie G. Ellis E. M. (2009). Enzymes involved in the metabolism of γ-hydroxybutyrate in SH-SY5Y cells: identification of an iron-dependent alcohol dehydrogenase ADHFe1. *Chem. Biol. Interact.* 178 283–287. 10.1016/j.cbi.2008.10.025 19013439

[B57] Macnicol P. K. Jacobsen J. V. (2001). Regulation of alcohol dehydrogenase gene expression in barley aleurone by gibberellin and abscisic acid. *Plant Physiol.* 111 533–539. 10.1034/j.1399-3054.2001.1110414.x 11299019

[B58] ManrÍquez D. El-Sharkawy I. Flores F. B. El-Yahyaoui F. Regad F. Bouzayen M. (2006). Two highly divergent alcohol dehydrogenases of melon exhibit fruit ripening-specific expression and distinct biochemical characteristics. *Plant Mol. Biol.* 61 675–685. 10.1007/s11103-006-0040-9 16897483

[B59] Matsukura S. Mizoi J. Yoshida T. Todaka D. Ito Y. Maruyama K. (2010). Comprehensive analysis of rice DREB2-type genes that encode transcription factors involved in the expression of abiotic stress-responsive genes. *Mol. Genet. Genomics* 283 185–196. 10.1007/s00438-009-0506-y 20049613

[B60] Matton D. P. Constable P. Brisson N. (1990). Alcohol dehydrogenase gene expression in potato following elicitor and stress treatment. *Plant Mol. Biol.* 14 775–783. 10.1007/BF00016510 2102855

[B61] McKie J. H. Jaouhari R. Douglas K. T. Goffner D. Feuillet C. Grima-Pettenati J. (1993). A molecular model for cinnamyl alcohol dehydrogenase, a plant aromatic alcohol dehydrogenase involved in lignification. *Biochim. Biophys. Acta* 1202 61–69. 10.1016/0167-4838(93)90063-W 8373826

[B62] Min T. Yin X. R. Shi Y. N. Luo Z. R. Yao Y. C. Grierson D. (2012). Ethylene-responsive transcription factors interact with promoters of ADH and PDC involved in persimmon (*Diospyros kaki*) fruit de-astringency. *J. Exp. Bot.* 63 6393–6405. 10.1093/jxb/ers296 23095993 PMC3504493

[B63] Moummou H. Tonfack L. B. Chervin C. Benichou M. Youmbi E. Giniese C. (2012). Functional characterization of SlscADH1, a fruit-ripening-associated short-chain alcohol dehydrogenase of tomato. *J. Plant Physiol.* 169 1435–1444. 10.1016/j.jplph.2012.06.007 22818888

[B64] Nian H. J. Meng Q. C. Zhang W. Chen L. M. (2013). Overexpression of the formaldehyde dehydrogenase gene from *Brevibacillus brevis* to enhance formaldehyde tolerance and detoxification of tobacco. *Appl. Biochem. Biotechnol.* 169 170–180. 10.1007/s12010-012-9957-4 23160947

[B65] Pathuri I. P. Reitberger I. E. Ckelhoven R. Proels R. K. (2011). Alcohol dehydrogenase 1 of barley modulates susceptibility to the parasitic fungus *Blumeria graminis* f.sp. hordei. *J. Exp. Bot.* 62 3449–3457. 10.1093/jxb/err017 21339386 PMC3130169

[B66] Peng H. P. Chan C. S. Shih M. C. Yang S. F. (2001). Signalling events in the hypoxic induction of alcohol dehydrogenase gene in *Arabidopsis*. *Plant Physiol.* 126 742–749. 10.1104/pp.126.2.742 11402202 PMC111164

[B67] Perry D. J. Furnier G. R. (1996). *Pinus banksiana* has at least seven expressed alcohol dehydrogenase genes in two linked groups. *Proc. Natl. Acad. Sci. U.S.A.* 93 13020–13023. 10.1073/pnas.93.23.13020 8917537 PMC24039

[B68] Persson B. Krook M. Jörnvall H. (1991). Characteristics of short-chain alcohol dehydrogenases and related enzymes. *Eur. J. Biochem.* 200 537–543. 10.1111/j.1432-1033.1991.tb16215.x 1889416

[B69] Plapp B. V. Lee A. T. Khanna A. Pryor J. M. (2013). Bradykinetic alcohol dehydrogenases make yeast fitter for growth in the presence of allyl alcohol. *Chem. Biol. Interact.* 202 104–110. 10.1016/j.cbi.2012.11.010 23200945 PMC3596495

[B70] Polichuk D. R. Zhang Y. S. Reed D. W. Schmidt J. F. Covello P. S. (2010). A glandular trichome-specific monoterpene alcohol dehydrogenase from *Artemisia annua*. *Phytochemistry* 711 1264–1269. 10.1016/j.phytochem.2010.04.026 20621795

[B71] Prashant S. Sunita M. S. L. Sirisha V. L. Bhaskar V. V. Rao A. M. Narasu M. L. (2012). Isolation of cinnamoyl CoA reductase and cinnamyl alcohol dehydrogenase gene promoters from *Leucaena leucocephala*, a leguminous tree species, and characterization of tissue-specific activity in transgenic tobacco. *Plant Cell Tissue Organ Cult.* 108 421–436. 10.1007/s11240-011-0053-1

[B72] Qi X. H. Xu X. W. Lin X. J. Zhang W. J. Chen X. H. (2012). Identification of differentially expressed genes in cucumber (*Cucumis sativus* L.) root under waterlogging stress by digital gene expression profile. *Genomics* 99 160–168. 10.1016/j.ygeno.2011.12.008 22240004

[B73] Quaglia D. Pori M. Galletti P. Emer E. Paradisi F. Giacomini D. (2013). His-tagged Horse Liver Alcohol Dehydrogenase: immobilization and application in the bio-based enantioselective synthesis of (S)-arylpropanols. *Process Biochem.* 48 810–818. 10.1016/j.procbio.2013.03.016

[B74] Rustérucci C. Espunya M. C. Díaz M. Chabannes M. Martínez M. C. (2007). S-nitrosoglutathione reductase affords protection against pathogens in *Arabidopsis*, both locally and systemically. *Plant Physiol.* 143 1282–1292. 10.1104/pp.106.091686 17277089 PMC1820916

[B75] Singh R. K. Sane V. A. Misra A. Ali S. A. Nath P. (2010). Differential expression of the mango alcohol dehydrogenase gene family during ripening. *Phytochemistry* 71 1485–1494. 10.1016/j.phytochem.2010.05.024 20598721

[B76] Skriver K. Mundy J. (1990). Gene expression in response to abscisic acid and osmotic stress. *Plant Cell* 2 503–512. 10.2307/3869112 2152172 PMC159906

[B77] Small R. L. Wendel J. F. (2000). Copy number lability and evolutionary dynamics of the Adh gene family in diploid and tetraploid cotton (Gossypium). *BMC Genet.* 155:1913–1926. 10.1093/genetics/155.4.1913 PMC1461218 10924485

[B78] Speirs J. Correll R. Cain P. (2002). Relationship between ADH activity, ripeness and softness in six tomato cultivars. *Sci. Hortic.* 93 137–142. 10.1016/S0304-4238(01)00316-8

[B79] Speirs J. Lee E. Holt K. Kim Y. D. Scott N. S. Loveys B. (1998). Genetic manipulation of alcohol deshydrogenase levels in ripening tomato fruit affects the balance of some flavour aldehydes and alcohols. *Plant Physiol.* 117 1047–1058. 10.1104/pp.117.3.1047 9662548 PMC34921

[B80] Srivastava A. Handa A. K. (2005). Hormonal regulation of tomato fruit development: a molecular perspective. *J. Plant Growth Regul.* 24 67–82. 10.1007/s00344-005-0015-0

[B81] Strommer J. (2011). The plant ADH gene family. *Plant J.* 66 128–142. 10.1111/j.1365-313X.2010.04458.x 21443628

[B82] Su Z. Ma X. Guo H. H. Sukiran N. L. Guo B. Assmann S. M. (2013). Flower development under drought stress: morphological and transcriptomic analyses reveal acute responses and long-term acclimation in *Arabidopsis*. *Plant Cell* 25 3785–3807. 10.1105/tpc.113.115428 24179129 PMC3877795

[B83] Sung C. K. Kim S. M. Oh C. J. Yang S. A. Han B. H. Mo E. K. (2012). Taraxerone enhances alcohol oxidation via increases of alcohol dehyderogenase (ADH) and acetaldehyde dehydrogenase (ALDH) activities and gene expressions. *Food Chem. Toxicol.* 50 2508–2514. 10.1016/j.fct.2012.04.031 22554647

[B84] Tesniere C. Pradal M. El-Kereamy A. Torregrosa L. Chatelet P. Roustan J. P. (2004). Involvement of ethylene signalling in a non-climacteric fruit: new elements regarding the regulation of ADH expression in grapevine. *J. Exp. Bot.* 55 2235–2240. 10.1093/jxb/erh244 15333642

[B85] Tesnière C. Verriès C. (2000). Molecular cloning and expression of cDNAs encoding alcohol dehydrogenases from *Vitis vinifera* L. during berry development. *Plant Sci.* 157 77–88. 10.1016/S0168-9452(00)00274-0 10940471

[B86] Thompson C. E. Fernandes C. L. de Souza O. N. de Freitas L. B. Salzano F. M. (2010). Evaluation of the impact of functional diversification on Poaceae, Brassicaceae, Fabaceae, and Pinaceae alcohol dehydrogenase enzymes. *J. Mol. Model.* 16 919–928. 10.1007/s00894-009-0576-0 19834749

[B87] Thompson C. E. Salzano F. M. de Souza O. N. Freitas L. B. (2007). Sequence and structural aspects of the functional diversification of plant alcohol dehydrogenases. *Gene* 396 108–115. 10.1016/j.gene.2007.02.016 17433574

[B88] Tonfack L. B. Moummou H. Latché A. Youmbi E. Benichou M. Pech J. C. (2011). The plant SDR superfamily: involvement in primary and secondary metabolism. *Curr. Top. Plant Biol.* 12 41–53.

[B89] Trainotti L. Tadiello A. Casadoro G. (2007). The involvement of auxin in the ripening of climacteric fruits comes of age: the hormone plays a role of its own and has an intense interplay with ethylene in ripening peaches. *J. Exp. Bot.* 58 3299–3308. 10.1093/jxb/erm178 17925301

[B90] Williamson V. M. Paquin C. E. (1987). Homology of *Saccharomyces cerevisiæ* ADH4 to an iron-activated alcohol dehydrogenase from *Zymomonas mobilis*. *Mol. Gen. Genet.* 209 374–381. 10.1007/BF00329668 2823079

[B91] Yamauchi T. Watanabe K. Fukazawa A. Mori H. Abe F. Kawaguchi K. (2014). Ethylene and reactive oxygen species are involved in root aerenchyma formation and adaptation of wheat seedlings to oxygen-deficient conditions. *J. Exp. Bot.* 65 261–273. 10.1093/jxb/ert371 24253196 PMC3883296

[B92] Zhang B. Xi W. P. Wei W. W. Shen J. Y. Ferguson I. Chen K. S. (2011). Changes in aroma-related volatiles and gene expression during low temperature storage and subsequent shelf-life of peach fruit. *Postharvest Biol. Technol.* 60 7–16. 10.1016/j.postharvbio.2010.09.012

[B93] Zhang D. Lopez-Reyes J. G. Spadaro D. Garibaldi A. Gullino M. L. (2010). Efficacy of yeast antagonists used individually or in combination with hot water dipping for control of postharvest brown rot of peaches. *J. Plant Dis. Prot.* 117 226–232. 10.1007/BF03356365

[B94] Zheng X. Y. Hu C. Y. Spooner D. Liu J. Cao J. S. Teng Y. W. (2011). Molecular evolution of Adh and LEAFY and the phylogenetic utility of their introns in *Pyrus* (Rosaceae). *BMC Evol. Biol.* 11:255 10.1186/1471-2148-11-255 PMC3182939 21917170

