# Supplementary material for: The Alcohol Dehydrogenase Gene Family in Melon (Cucumis melo L.): Bioinformatic Analysis and Expression Patterns
Source: Front Plant Sci. 2016 May 18;7:670. doi: 10.3389/fpls.2016.00670 (PMC4870255; doi:10.3389/fpls.2016.00670)
Supplement: FIGURE S1 — Phylogenetic tree of melon medium-chain ADH proteins. The amino acid sequences were aligned by the Clustal Omega program, and the neighborjoining tree was drawn with TreeView. The corresponding GenBank and the melon genome (https://melonomics.net/) were noted in the phylogenetic tree and the accession number in the melon genome were CmADH1 (MELO3C023685P4), CmADH3 (MELO3C026552P1), CmADH4 (MELO3C027151P1), CmADH5 (MELO3C00579 2P1), CmADH6 (MELO3C026553P1), CmADH7 (MELO3C002189P1), CmADH8 (ME LO3C003251P1), CmADH9 (MELO3C011043P1), CmADH10 (MELO3C026554P2), CmADH11 (MELO3C023687P1), and CmFDH1 (MELO3C022399P1). The number for each interior branch was the percentage of bootstraps value (1000 replicates). Black circle denoted 13 CmADHs. ADHs from other plants in our paper are in Supplementary Table S3. [file Presentation_1.ZIP › Supporting Information/Figure S13 .pptx]

## Slide 1
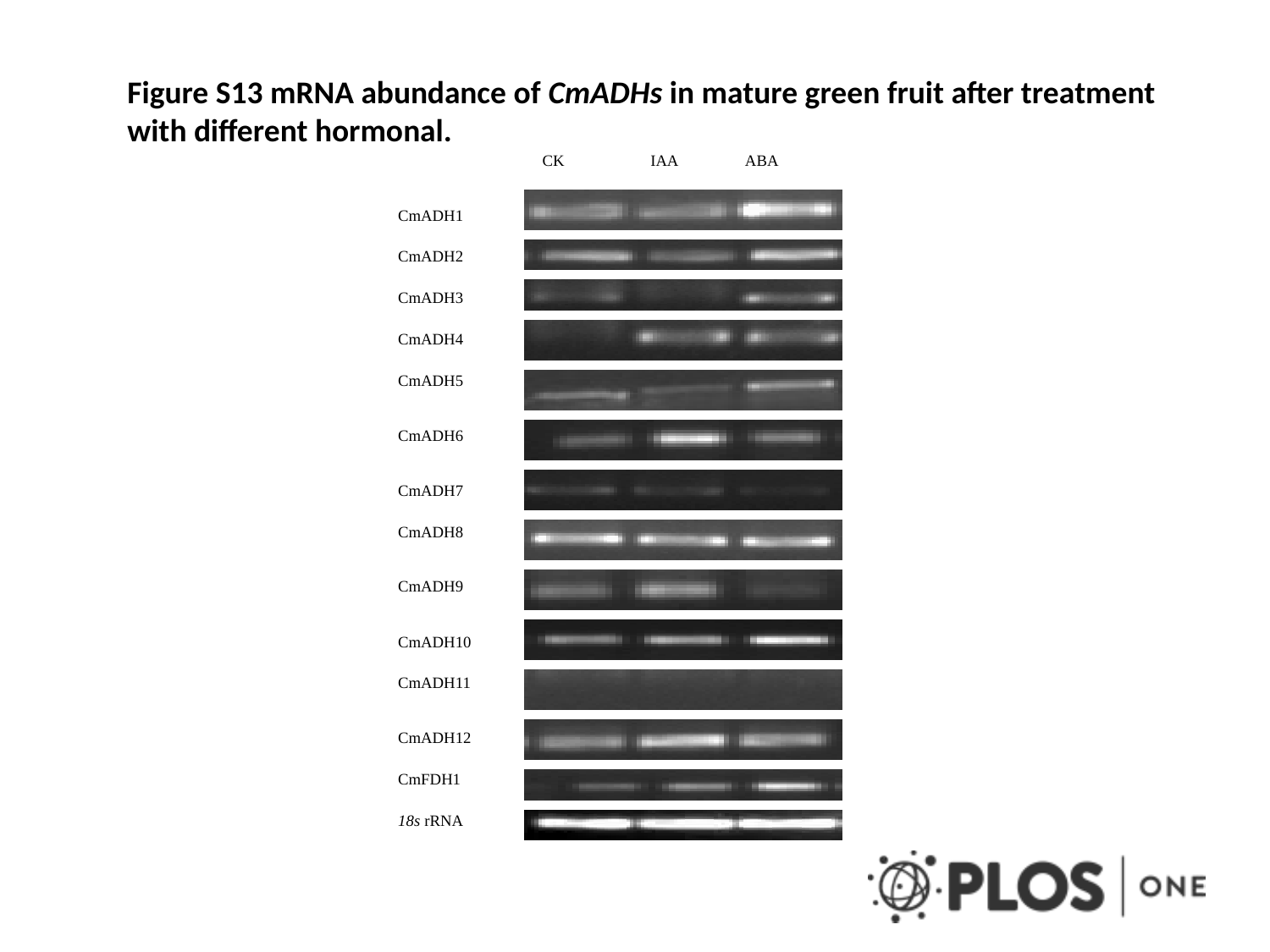

Figure S13 mRNA abundance of CmADHs in mature green fruit after treatment with different hormonal.
CK
IAA
ABA
CmADH1
CmADH2
CmADH3
CmADH4
CmADH5
CmADH6
CmADH7
CmADH8
CmADH9
CmADH10
CmADH11
CmADH12
CmFDH1
18s rRNA
