# Supplementary material for: The Alcohol Dehydrogenase Gene Family in Melon (Cucumis melo L.): Bioinformatic Analysis and Expression Patterns
Source: Front Plant Sci. 2016 May 18;7:670. doi: 10.3389/fpls.2016.00670 (PMC4870255; doi:10.3389/fpls.2016.00670)
Supplement: FIGURE S1 — Phylogenetic tree of melon medium-chain ADH proteins. The amino acid sequences were aligned by the Clustal Omega program, and the neighborjoining tree was drawn with TreeView. The corresponding GenBank and the melon genome (https://melonomics.net/) were noted in the phylogenetic tree and the accession number in the melon genome were CmADH1 (MELO3C023685P4), CmADH3 (MELO3C026552P1), CmADH4 (MELO3C027151P1), CmADH5 (MELO3C00579 2P1), CmADH6 (MELO3C026553P1), CmADH7 (MELO3C002189P1), CmADH8 (ME LO3C003251P1), CmADH9 (MELO3C011043P1), CmADH10 (MELO3C026554P2), CmADH11 (MELO3C023687P1), and CmFDH1 (MELO3C022399P1). The number for each interior branch was the percentage of bootstraps value (1000 replicates). Black circle denoted 13 CmADHs. ADHs from other plants in our paper are in Supplementary Table S3. [file Presentation_1.ZIP › Supporting Information/Figure S4.pptx]

## Slide 1
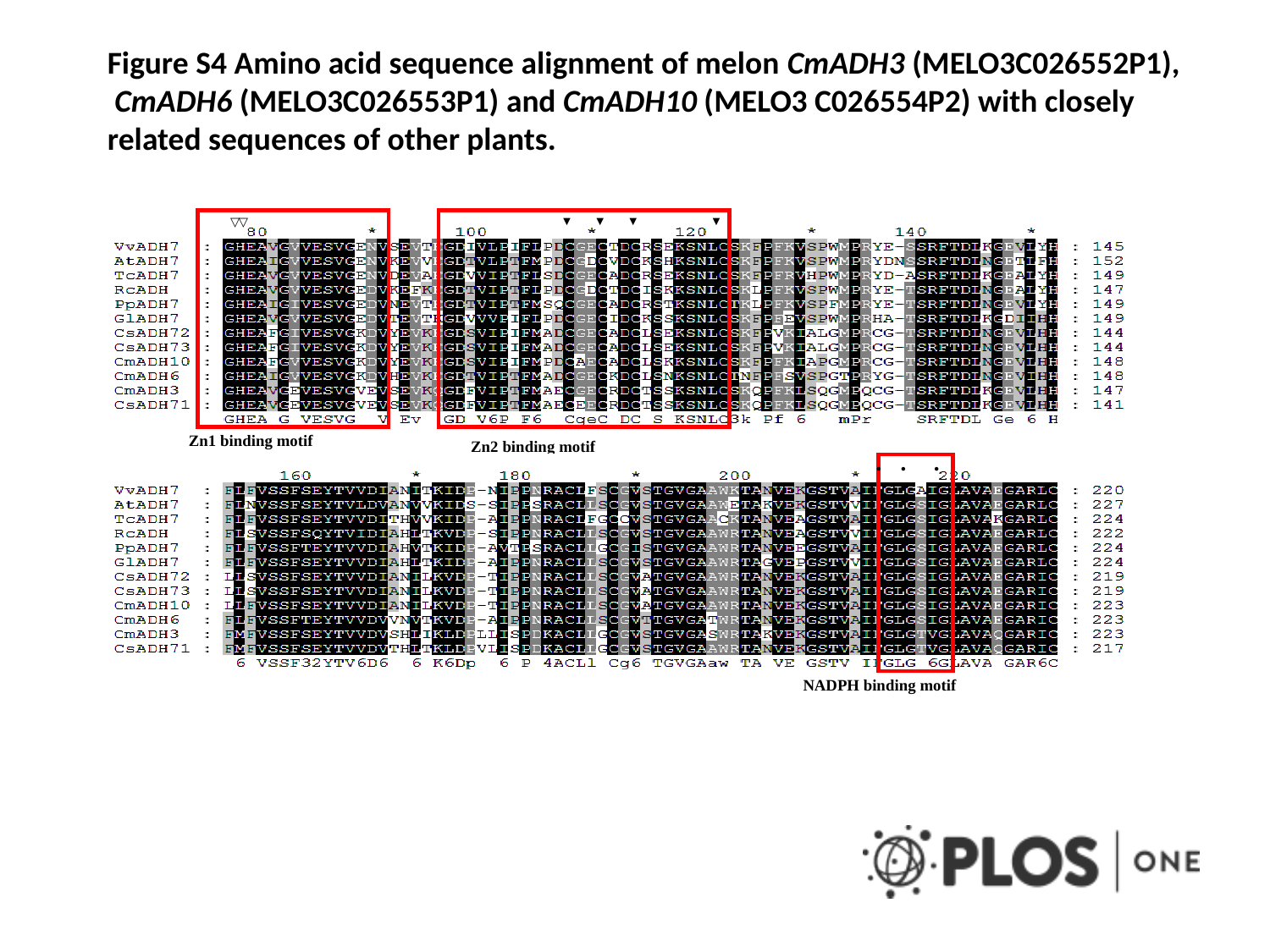

Figure S4 Amino acid sequence alignment of melon CmADH3 (MELO3C026552P1), CmADH6 (MELO3C026553P1) and CmADH10 (MELO3 C026554P2) with closely related sequences of other plants.
▽
▽
▼
▼
▼
▼
Zn1 binding motif
Zn2 binding motif
●
●
●
NADPH binding motif
