# Supplementary material for: The Alcohol Dehydrogenase Gene Family in Melon (Cucumis melo L.): Bioinformatic Analysis and Expression Patterns
Source: Front Plant Sci. 2016 May 18;7:670. doi: 10.3389/fpls.2016.00670 (PMC4870255; doi:10.3389/fpls.2016.00670)
Supplement: FIGURE S1 — Phylogenetic tree of melon medium-chain ADH proteins. The amino acid sequences were aligned by the Clustal Omega program, and the neighborjoining tree was drawn with TreeView. The corresponding GenBank and the melon genome (https://melonomics.net/) were noted in the phylogenetic tree and the accession number in the melon genome were CmADH1 (MELO3C023685P4), CmADH3 (MELO3C026552P1), CmADH4 (MELO3C027151P1), CmADH5 (MELO3C00579 2P1), CmADH6 (MELO3C026553P1), CmADH7 (MELO3C002189P1), CmADH8 (ME LO3C003251P1), CmADH9 (MELO3C011043P1), CmADH10 (MELO3C026554P2), CmADH11 (MELO3C023687P1), and CmFDH1 (MELO3C022399P1). The number for each interior branch was the percentage of bootstraps value (1000 replicates). Black circle denoted 13 CmADHs. ADHs from other plants in our paper are in Supplementary Table S3. [file Presentation_1.ZIP › Supporting Information/Table S1.docx]

Table S1: List and description of nucleotide motifs discovered in promoter region of melon ADH genes

| Motif name | Gene name | Motif sequence | Motif database | Motif function |
| --- | --- | --- | --- | --- |
| TC-rich repeats | *CmADH1* | ATTTTCTTCA;  GTTTTCTTAC | PlantCARE | Cis-acting element involved in defense and stress responsiveness |
|  | *CmADH2* |  |  |  |
|  | *CmADH3* |  |  |  |
|  | *CmADH5* |  |  |  |
|  | *CmADH6* |  |  |  |
|  | *CmADH7* |  |  |  |
|  | *CmADH10* |  |  |  |
| BOX-W1(W BOX) | *CmADH2* | TTGACC | PlantCARE | Fungal elicitor responsive element |
|  | *CmADH3* |  |  |  |
|  | *CmADH5* |  |  |  |
|  | *CmADH6* |  |  |  |
|  | *CmADH7* |  |  |  |
|  | *CmADH10* |  |  |  |
|  | *CmADH11* |  |  |  |
| ARE | *CmFDH1* | TGGTTT | PlantCARE | Cis-acting element involved in hypoxia stress responsiveness |
|  | *CmADH1* |  |  |  |
|  | *CmADH3* |  |  |  |
|  | *CmADH5* |  |  |  |
|  | *CmADH6* |  |  |  |
|  | *CmADH7* |  |  |  |
|  | *CmADH8* |  |  |  |
|  | *CmADH10* |  |  |  |
|  | *CmADH11* |  |  |  |
|  | *CmADH12* |  |  |  |
| CGTCA-motif | *CmFDH1*  *CmADH1*  *CmADH2*  *CmADH6*  *CmADH7*  *CmADH9*  *CmADH12* | CGTCA, TGACG | Plant CARE | Cis-acting regulatory element involved in the MeJA-responsiveness |
| TGACG-motif | *CmFDH1*  *CmADH1*  *CmADH2*  *CmADH3*  *CmADH6*  *CmADH7*  *CmADH8*  *CmADH9*  *CmADH12* | TGACG | Plant CARE | Cis-acting regulatory element involved in the MeJA-responsiveness |
| ELI-box3 | *CmADH9* | AAACCAATT | PlantCARE | Elicitor responsive element |
| AT-rich sequence | *CmFDH1*  *CmADH3* | TAAAATACT | PlantCARE | Elicitor responsive element |
| TA-rich region | *CmADH9* |  | PlantCARE | enhancer |
| CCAAT-box | *CmADH3* | CAACGG | Plant CARE | Cis-acting regulatory element involved in the MeJA-responsiveness |
|  | *CmADH5* |  |  |  |
|  | *CmADH9* |  |  |  |
| HSE | *CmADH1* | AAAAAATTTC | PlantCARE | Cis-acting element involved in heat stress responsiveness |
|  | *CmADH2* | AAAAAATTTC |  |  |
|  | *CmADH3* | AGAAAATTCG |  |  |
|  | *CmADH5* | AAAATTTAAT |  |  |
|  | *CmADH6* |  |  |  |
|  | *CmADH8* |  |  |  |
|  | *CmADH9* |  |  |  |
|  | *CmADH10* | AAAAAATGTC |  |  |
| MBS-Ⅰ | *CmADH3* | AAAAAAC; GTTA | PlantCARE | MYB binding site involved in flavonoid biosynthesis |
|  | *CmADH6* |  |  |  |
| MBS-Ⅱ | *CmADH9* | AAAAGTTAGTTA | PlantCARE | MYB binding site involved in flavonoid biosynthesis |
| LTR | *CmADH1* | CCGAAA | PlantCARE | Cis-acting element involved in Low Temperature responsiveness |
|  | *CmADH2* |  |  |  |
|  | *CmADH5* |  |  |  |
|  | *CmADH10* |  |  |  |
| MBS | *CmFDH1* | CAACTG | PlantCARE | MYB binding site involved in drought-inducibility |
|  | *CmADH3* |  |  |  |
|  | *CmADH5* |  |  |  |
|  | *CmADH6* |  |  |  |
|  | *CmADH7* |  |  |  |
|  | *CmADH9* |  |  |  |
|  | *CmADH12* |  |  |  |
| GARE-motif | *CmADH1* | TCTGTTG | PlantCARE | Cis-acting element involved in the gibberellin responsiveness |
|  | *CmADH9* |  |  |  |
|  |  |  |  |  |
|  |  |  |  |  |
| P-box | *CmADH1* | CCTTTTG | PlantCARE | Cis-acting element involved in the gibberellin responsiveness |
|  | *CmADH10* |  |  |  |
| ABRE | *CmFDH1* | ACGTGGC | PlantCARE | Cis-acting element involved in the abscisic acid responsiveness |
|  | *CmADH2* | ACGTGGC |  |  |
|  | *CmADH3* | CGCACGTGTC |  |  |
|  | *CmADH10* | CACGTG |  |  |
| SARE | *CmADH3* | TTCGACCATCTT | PlantCARE | Cis-acting regulatory element involved in the SA-responsiveness |
| EIRE | *CmADH3* | TTCGACC | PlantCARE | Elicitor responsive element |
|  | *CmADH11* |  |  |  |
| ERE | *CmFDH1* | ATTTCAAA | PlantCARE | Ethylene-responsive |
|  | *CmADH1* |  |  |  |
|  | *CmADH2* |  |  |  |
|  | *CmADH3* |  |  |  |
|  | *CmADH5* |  |  |  |
|  | *CmADH6* |  |  |  |
|  | *CmADH8* |  |  |  |
|  | *CmADH12* |  |  |  |
| TGA | *CmADH6* | AACGAC  AACGAC；TGACGTAA  AACGAC | PlantCARE | Auxin responsive |
|  | *CmADH9* |  |  |  |
|  | *CmADH12* |  |  |  |
| TCA-element | *CmFDH1* | CCATCTTTTT | PlantCARE | Salicylic acid response |
|  | *CmADH1* |  |  |  |
|  | *CmADH3* |  |  |  |
|  | *CmADH5* |  |  |  |
|  | *CmADH7* |  |  |  |
|  | *CmADH8* |  |  |  |
|  | *CmADH9* | TCAGAAGAGA |  |  |
|  | *CmADH12* | TCAGAAAAGG |  |  |
