# Supplementary material for: The Alcohol Dehydrogenase Gene Family in Melon (Cucumis melo L.): Bioinformatic Analysis and Expression Patterns
Source: Front Plant Sci. 2016 May 18;7:670. doi: 10.3389/fpls.2016.00670 (PMC4870255; doi:10.3389/fpls.2016.00670)
Supplement: FIGURE S1 — Phylogenetic tree of melon medium-chain ADH proteins. The amino acid sequences were aligned by the Clustal Omega program, and the neighborjoining tree was drawn with TreeView. The corresponding GenBank and the melon genome (https://melonomics.net/) were noted in the phylogenetic tree and the accession number in the melon genome were CmADH1 (MELO3C023685P4), CmADH3 (MELO3C026552P1), CmADH4 (MELO3C027151P1), CmADH5 (MELO3C00579 2P1), CmADH6 (MELO3C026553P1), CmADH7 (MELO3C002189P1), CmADH8 (ME LO3C003251P1), CmADH9 (MELO3C011043P1), CmADH10 (MELO3C026554P2), CmADH11 (MELO3C023687P1), and CmFDH1 (MELO3C022399P1). The number for each interior branch was the percentage of bootstraps value (1000 replicates). Black circle denoted 13 CmADHs. ADHs from other plants in our paper are in Supplementary Table S3. [file Presentation_1.ZIP › Supporting Information/Table S2.docx]

Table S2 CmADH subcellular localization prediction

| ADH | LocTree3 | | CELLOV 2.5 | | Pslpre | | | Wolfprort | |
| --- | --- | --- | --- | --- | --- | --- | --- | --- | --- |
|  | Score | Localization Class | Score | Localization Class | RI | Expected Accuracy | Localization Class | Score | Localization Class |
| CmFDH1 | 74 | cytoplasm | 2.882 | cytoplasm | 2 | 68.1% | cytoplasm | 12 | cytoplasm |
| CmADH1 | 84 | cytoplasm | 3.821 | cytoplasm | 3 | 71.1% | cytoplasm | 8 | cytoplasm |
| CmADH2 | 12 | peroxisome | 3.537 | cytoplasm | 4 | 90.2% | cytoplasm | 9 | cytoplasm |
| CmADH3 | 38 | cytoplasm | 3.587 | cytoplasm | 1 | 53.1 | cytoplasm | 8 | cytoplasm |
| CmADH4 | 83 | cytoplasm | 3.732 | cytoplasm | 4 | 90.2 | cytoplasm | 13 | cytoplasm |
| CmADH5 | 41 | cytoplasm | 3.960 | cytoplasm | 3 | 71.1% | cytoplasm | 5 | cytoplasm |
| CmADH6 | 39 | cytoplasm | 3.379 | cytoplasm | 3 | 71.1% | cytoplasm | 13 | cytoplasm |
| CmADH7 | 43 | cytoplasm | 2.130 | OuterMembrane | 2 | 68.1% | cytoplasm | 6 | chloroplast |
| CmADH8 | 19 | cytoplasm | 4.055 | cytoplasm | 3 | 71.1% | cytoplasm | 7 | mitochondrial |
| CmADH9 | 37 | cytoplasm | 4.566 | cytoplasm | 3 | 71.1% | cytoplasm | 6 | cytoplasm |
| CmADH10 | 36 | cytoplasm | 3.574 | cytoplasm | 2 | 68.1% | cytoplasm | 7 | cytoplasm |
| CmADH11 | 66 | cytoplasm | 4.089 | cytoplasm | 3 | 71.1% | cytoplasm | 14 | cytoplasm |
| CmADH12 | 34 | peroxisome | 1.777 | cytoplasm | 2 | 68.1% | cytoplasm | 5 | chloroplast |
